# SLC5A3 depletion promotes apoptosis by inducing mitochondrial dysfunction and mitophagy in gemcitabine-resistant pancreatic cancer cells

**DOI:** 10.1038/s41419-025-07476-5

**Published:** 2025-03-07

**Authors:** Minsoo Kim, Woosol Chris Hong, Hyeon Woong Kang, Ju Hyun Kim, Dongyong Lee, Jae-Ho Cheong, Hye-Sol Jung, Wooil Kwon, Jin-Young Jang, Hyo Jung Kim, Joon Seong Park

**Affiliations:** 1 Brain Korea 21 PLUS Project for Medical Science, Yonsei University, College of Medicine, Seoul, Republic of Korea; 2 Department of Medicine, Yonsei University College of Medicine, Seoul, Republic of Korea; 3 Department of Surgery and Cancer Research Institute, Seoul National University College of Medicine, Seoul, Republic of Korea; 4 Biomedical Research Institute, Seoul National University Hospital, Seoul, Republic of Korea

**Keywords:** Tumour biomarkers, Predictive markers

## Abstract

Pancreatic ductal adenocarcinoma (PDAC) is a highly aggressive cancer with poor prognosis, largely due to the rapid development of chemoresistance in patients. Mitochondrial dynamics play a crucial role in cancer cell survival. Currently, the specific mechanisms underlying gemcitabine resistance in PDAC remain unknown. In this study, we identified the sodium/myo-inositol co-transporter solute carrier family 5 member 3 (SLC5A3) as a key modulator promoting chemoresistance in PDAC. SLC5A3 levels were significantly upregulated in gemcitabine-resistant PDAC cells, enhancing their cell survival by stabilizing the mitochondrial functions and inhibiting apoptosis. Mitochondrial analysis showed that SLC5A3 inhibition disrupted the mitochondrial dynamics, leading to increased reactive oxygen species production, mitochondrial fission, and impaired oxidative phosphorylation. Moreover, SLC5A3 inhibition activated the PTEN-induced kinase 1/Parkin-mediated mitophagy pathway, resulting in the excessive removal of damaged and healthy mitochondria, thereby depleting the mitochondrial reserves and sensitizing the cells to apoptosis. In vivo studies revealed that targeting SLC5A3 enhanced the efficacy of gemcitabine and significantly reduced the tumor growth. Collectively, these results suggest SLC5A3-mediated mitochondrial regulation as a promising therapeutic strategy to overcome gemcitabine resistance in PDAC.

## Introduction

Pancreatic ductal adenocarcinoma (PDAC) is the most common type of malignant pancreatic cancer, with a 5-year overall survival rate of only 13% [1]. Owing to the aggressive nature of the disease and limited treatment options, gemcitabine is often used as the primary chemotherapeutic agent for PDAC [2]. However, rapid development of drug resistance often limits its efficacy. Various mechanisms of gemcitabine resistance in PDAC, such as the activation of pro-survival pathways, altered drug metabolism, and disruption of apoptosis, have been identified [3–6]. Although these mechanisms provide some insights, the specific cellular processes and key regulators responsible for gemcitabine resistance in PDAC remain unclear. Therefore, resistance mechanisms and novel biomarkers should be explored to improve the treatment outcomes and prognosis of patients with PDAC.

Mitochondria are crucial for cell energy production and apoptosis regulation and play an important role in the induction of drug resistance in cancer [7–9]. Balance between mitochondrial fusion and fission helps tumor cells meet their energy demands [10]. Disruption of this balance renders mitochondria more vulnerable to oxidative stress, leading to the activation of the mitophagy pathway mediated by PTEN-induced kinase 1 (PINK1) and E3 ubiquitin ligase Parkin [11, 12]. Although mitophagy typically only removes the damaged mitochondria, excessive mitophagy also depletes the healthy mitochondria, thereby decreasing energy production [13–16]. Energy deficiency renders the cancer cells more susceptible to apoptosis. Therefore, understanding the regulation of mitochondrial dynamics and mitophagy is essential to develop therapeutic strategies against drug resistance in cancer.

Solute carrier (SLC) transporters, the largest family of transmembrane transporters, are crucial for cellular metabolism regulation and have been implicated in various cancers, including breast, lung and prostate cancers [17–19]. Recent studies have revealed the roles of SLC transporters in tumors, particularly pancreatic cancer [20]. Specifically, SLC family 5 member 3 (SLC5A3) is involved in several cancers. SLC5A3 plays key roles in cancer cell survival and proliferation via cellular osmoregulation and metabolic demand regulation [21]. SLC5A3 promotes cell survival via myo-inositol metabolism in acute myeloid leukemia [22]. It drives tumor progression by activating the Akt–mTOR pathway in non-small cell lung cancer [23]. Additionally, SLC5A3 expression is upregulated via TonEBP binding, contributing to tumor growth in cervical cancer [24]. Although the oncogenic roles of SLC5A3 have been explored in different cancers, its specific functions and association with chemoresistance in pancreatic cancer remain unknown. Therefore, investigating the roles of SLC5A3 in chemoresistance will help to overcome chemoresistance in pancreatic cancer, thereby improving the patient outcomes.

In this study, SLC5A3 expression was elevated in gemcitabine-resistant PDAC, contributing to the inhibition of apoptosis and enhancement of gemcitabine resistance. SLC5A3 inhibition caused mitochondrial dysfunction by increasing the reactive oxygen species (ROS) levels, reducing oxidative phosphorylation (OXPHOS), and inducing apoptosis. Additionally, it disrupted mitochondrial dynamics and enhanced mitophagy, leading to the selective removal of damaged mitochondria. This excessive mitophagy further depleted the healthy mitochondria, thereby decreasing cellular energy production and sensitizing the cancer cells to apoptosis. Therefore, targeting SLC5A3 and modulating mitophagy are promising strategies to overcome gemcitabine resistance in PDAC.

## Materials and Methods

### Patient tissue collection

This study adhered to the ethical principles of the Declaration of Helsinki and was approved by the Institutional Review Boards (IRB) of the Gangnam Severance Hospital (IRB No. 3-2021-0414) and Seoul National University Hospital (IRB No. H-1705-031-852). Tissue samples were collected from patients with PDAC and individuals with normal pancreatic tissues who underwent surgical resection or biopsy at the Gangnam Severance Hospital.

### Patient selection

Patients diagnosed with PDAC at the Gangnam Severance Hospital between 2018 and 2019 underwent pancreatic resection. All patients received adjuvant gemcitabine chemotherapy (1000 mg/m²) over 30 min once a week for 3–4 weeks over six cycles. Patients showing no recurrence up to six months post-chemotherapy were classified as gemcitabine-sensitive, whereas those showing recurrence were classified as gemcitabine-resistant. Written informed consent was obtained from all participants prior to their inclusion in the study.

### Kaplan–Meier survival analysis

Kaplan–Meier survival analysis was conducted using data from the TCGA–PAAD dataset and the SNU patient cohort. Patients were stratified into “high” and “low” SLC5A3 expression groups based on optimal cut-off values using the X-tile software (Yale University, New Haven, CT, USA) (version 3.6.1). Kaplan–Meier survival curves were generated using GraphPad Prism software (version 10.3.0).

### Clustered heatmap analysis

To investigate the relationship between SLC5A3 expression and other genes, Pearson correlation coefficients were calculated. The genes were then ranked based on their correlation coefficients in descending order. The top 50 genes most highly correlated with SLC5A3 were selected for further analysis.

### Cell culture

Human pancreatic cancer cell lines (AsPC-1, BxPC-3, Capan-1, PANC-1, and MIA PaCa-2) were purchased from the American Type Culture Collection (Manassas, VA, USA). AsPC-1, BxPC-3, and Capan-1 cells were cultured in the Roswell Park Memorial Institute-1640 medium (Biowest, Riverside, MO, USA), whereas PANC-1 and MIA PaCa-2 cells were cultured in the Dulbecco’s modified Eagle’s medium (Biowest) supplemented with 10% fetal bovine serum (Biowest) and 1% antibiotic–antimycotic (Gibco, Waltham, MA, USA). The cells were incubated at 37 °C in a humidified atmosphere containing 5% CO_2_. Gemcitabine-resistant cell lines were generated by exposing the parental cell lines to increasing concentrations of gemcitabine (0.01 μM, 0.05 μM, 0.1 μM, 0.5 μM) (Yuhan, Seoul, Korea) for three months, after which the PANC-1/GR cell line was continuously cultured in a medium containing 0.5 μM gemcitabine.

### Water-soluble tetrazolium salt (WST)-1 cell proliferation assay

WST-1 assay was performed as previously described [25]. Briefly, the cells were plated in a 96-well plate (5 × 10^3^ cells/well). After treatment with gemcitabine for 72 h, EZ-Cytox (DoGenBio, Seoul, Korea) was added to the cells for 1 h. Optical density was measured at 450 nm using the VersaMax Microplate Reader (Molecular Devices, San Jose, CA, USA). Half-maximal inhibitory concentration (IC_50_) values were calculated using the GraphPad Prism software version 10.3.0 (GraphPad Software, Boston, MA, USA).

### Wound healing assay

Wound-healing assays were conducted as previously described [25]. Briefly, the cells were plated in a 12-well plate (2 × 10^5^ cells/well), and linear wounds were created using a pipette tip. Microscopic images were captured at the indicated time points (0H, 16H, 24H), and wound area was quantified using the ImageJ software (NIH, Bethesda, MD, USA).

### Myo-inositol detection

PANC-1 cells were seeded in a 6-well plate (1.5 × 10^5^ cells/well) for myo-inositol detection using the Myo-Inositol Assay Kit (Abcam, Cambridge, UK), following the manufacturer’s protocol. Samples, standards, and background controls were prepared, and fluorescence was measured in endpoint mode at excitation/emission wavelengths of 535/587 nm using the VersaMax Microplate Reader (Molecular Devices).

### BrdU Incorporation Assay

PANC-1 cells were seeded in a 6-well plate (1.5 × 10^5^ cells/well) and incubated with BrdU (Sigma-Aldrich, St. Louis, MO, USA) at 37 °C for 1 h. Following incubation, cells were fixed with 70% ethanol for 1 h at room temperature and permeabilized with 0.2% Triton X-100 (Sigma-Aldrich) for 15 min. To denature the DNA, cells were treated with 2 N HCl (Duksan, Seoul, Korea) at 37 °C for 20 min, followed by blocking with 3% BSA (BOVOGEN Biologicals, Keilor East, Australia) at room temperature for 30 min. After washing with PBS, cells were incubated with primary antibodies at room temperature for 1 h, followed by incubation with the Alexa Fluor 488 goat anti-rat IgG (H + L) (Invitrogen, Waltham, MA, USA) secondary antibodies at room temperature for 30 min. Finally, cells were incubated with propidium iodide (Sigma-Aldrich) and RNase A (Sigma-Aldrich) for 30 min. Fluorescence was quantified using the FACSCanto II flow cytometer (BD Biosciences, Franklin Lakes, NJ, USA). Data analysis was conducted using the FlowJo software version 10.8.1 (FlowJo Software, Ashland, OR, USA).

### Cell cycle assay

Cell cycle analysis was performed as previously described [26]. Briefly, PANC-1 cells were fixed with 70% ethanol at 4 °C for 1 h, followed by incubation with propidium iodide (Sigma-Aldrich) and RNase A (Sigma-Aldrich) for 30 min. Fluorescence was quantified using the FACSCanto II flow cytometer (BD Biosciences). Data analysis was conducted using the FlowJo software version 10.8.1.

### Annexin V assay

FITC Annexin V Apoptosis Detection Kit I (BD Biosciences) was used to detect apoptosis, following the manufacturer’s protocol. Cells (1.5 × 10^5^ cells/well) were seeded in a 6-well plate, transfected with a small interfering RNA (Santa Cruz Biotechnology, Dallas, TX, USA) and incubated for 48 h. After staining with FITC Annexin V and propidium iodide, fluorescence intensity was quantified using the FACSCanto II flow cytometer (BD Biosciences). Data analysis was performed using the FlowJo software version 10.8.1.

### TdT-mediated dUTP nick-end labeling (TUNEL) assay

TUNEL assay was performed using the TUNEL Assay Kit (Abcam), according to the manufacturer’s instructions. The PANC-1 cells (1.5 × 10^4^ cells/well) were seeded in a confocal plate (SPL Life Science, Pocheon, Korea), fixed with 4% paraformaldehyde (CELLNEST, Hanam, Korea) at 4 °C for 15 min, and permeabilized with 0.2% Triton X-100 (Sigma-Aldrich) for 5 min. Fluorescent images were captured using the Zeiss LSM980 confocal microscope (Zeiss, Oberkochen, Germany).

### Measurement of ROS levels

ROS levels were measured via 2′,7′-dichlorofluorescein diacetate staining (Sigma-Aldrich). PANC-1 cells (1.5 × 10^5^ cells/well) were seeded in a 6-well plate and incubated with 2′,7′-dichlorofluorescein diacetate at 37 °C for 30 min. Fluorescence intensity was measured using the FACSCanto II flow cytometer, and data were analyzed using the FlowJo software version 10.8.1.

### Isolation of mitochondria

Mitochondrial and cytosolic fractions were isolated from 2 × 10^7^ PANC-1 cells using the Mitochondria Isolation Kit for Cultured Cells (Thermo Fisher Scientific, Waltham, MA, USA), following the manufacturer’s instructions.

### Mitochondrial stress test

Oxygen consumption rate (OCR) was measured using the Seahorse XFp Analyzer (Agilent, Santa Clara, CA, USA) with the Seahorse XFp Cell Mito Stress Test Kit (Agilent), as per the manufacturer’s instructions. Briefly, PANC-1 cells (1.5 × 10^5^ cells/well) were transfected with siRNAs and incubated in the XF assay medium at 37 °C for 1 h. Then, oligomycin (2 µM), FCCP (2 µM), and rotenone/antimycin A (1 µM) (Agilent) were sequentially injected, and OCR was normalized to the total cell number.

### Real-time ATP production rate assay

Real-time ATP production rates were measured using the Seahorse XFe96 Analyzer (Agilent) with the Seahorse XF Real-Time ATP Rate Assay Kit (Agilent), following the manufacturer’s instructions. The cells (5 × 10^3^ cells/well) were seeded in the Seahorse XF cell culture microplate and incubated in the Seahorse XF Dulbecco’s modified Eagle’s medium supplemented with 10 mM glucose, 1 mM pyruvate, and 2 mM glutamine. Oligomycin (1.5 µM) and rotenone/antimycin A (0.5 µM) were injected sequentially, and real-time ATP production rates were normalized to the total cell number.

### High-content screening

PANC-1 cells were seeded in a 96-well CellCarrier Ultra imaging plate (PerkinElmer, Waltham, MA, USA) and stained with 4′,6-diamidino-2-phenylindole and MitoTracker Red CMXRos (Cell Signaling Technology, Danvers, MA, USA). Images were acquired using Operetta CLS (PerkinElmer) with 20× and 40× objectives. Single-cell classification was performed using the PhenoLOGIC machine learning module in the Harmony Analysis Software version 4.5 (PerkinElmer).

### Transmission electron microscopy (TEM)

PANC-1 cells (1 × 10^6^) were fixed with 2.5% glutaraldehyde (Sigma-Aldrich) and prepared for TEM imaging following a series of steps, including dehydration, embedding, and sectioning with an Ultramicrotome (Leica Microsystems, Wetzlar, Germany), and polymerization in an oven processor. Glass knives produced using a glass knife maker (RMC Boeckeler, Tucson, AZ, USA) were used to obtain ultrathin sections. The sections were then imaged using a JEM-1400 Flash TEM (JEOL, Tokyo, Japan) at 120 kV at the Seoul National University Hospital Biomedical Research Institute, Seoul, Korea.

### Establishment of an orthotopic mouse model

Six-week-old male BALB/c nude mice were obtained from Orient Bio (Seongnam, Korea). PANC-1 SEN/RES cells (2.5 × 10^6^) were mixed with phosphate-buffered saline (Biowest) and matrigel (Corning, Bedford, MA, USA) and injected into the pancreas. Tumor growth was monitored, and SLC5A3 shRNA lentiviral particles (Origene, Rockville, MD, USA) were intravenously injected into the mice. After one week, gemcitabine (10 mg/kg) and carbonyl cyanide 3-chlorophenylhydrazone (CCCP; 3 mg/kg) were intraperitoneally injected twice per week. All procedures were approved by the Institutional Animal Care and Use Committee of the Yonsei Pharmaceutical University Experimental Animal Center, Seoul, Korea (approval #2022-0061).

### Immunohistochemical staining

Serial sections (5 µm) of each tissue block were adhered to poly-L-lysine-coated slides and incubated at 62 °C for 1 h. After deparaffinization with Neo-Clear (Sigma-Aldrich) and rehydration using a graded ethanol series, the sections were heated with Target Retrieval Solution Citrate pH 6 (Dako, Glostrup, Denmark) for 30 min and blocked with the IgG Fab fragment (Santa Cruz Biotechnology, Dallas, TX, USA) and 10% Normal Goat Serum (Jackson ImmunoResearch, West Grove, PA, USA) in PBS for 1 h. After washing with PBS, the slides were incubated with primary antibodies overnight at 4 °C, followed by incubation with the Alexa Fluor 488 goat anti-mouse IgG (H + L) (Invitrogen) and Cy5 goat anti-rabbit IgG (H + L) (Invitrogen) secondary antibodies. Finally, the slides were mounted with the Fluoroshield Mounting Medium containing 4′,6-diamidino-2-phenylindole (Abcam) to preserve the tissues for microscopic analysis. Finally, the slides were observed under the TCS SP8 STED CW confocal laser-scanning microscope (Leica Microsystems, Wetzlar, Germany).

### Real-time polymerase chain reaction (PCR)

Total RNA was extracted from the PANC-1 cells and tissue samples using the TRIzol reagent (Invitrogen), according to the manufacturer’s protocol. cDNA was synthesized using 1 µg total RNA and Maxime RT PreMix (Intron, Seongnam, Korea). Real-Time PCR was conducted using the Power SYBR Green PCR Master Mix (Applied Biosystems, Waltham, MA, USA) with the 7500 Real-Time PCR System (Applied Biosystems). Gene expression was normalized to that of glyceraldehyde 3-phosphate dehydrogenase mRNA using the 2^−ΔΔC*t*^ method. The primer sequences for Real-Time PCR are listed in Supplementary Table 1.

### RNA sequencing and data analysis

Total RNA was extracted, and libraries were prepared using the QuantSeq 3′ mRNA-Seq Library Prep Kit FWD (Lexogen, Vienna, Austria), following the manufacturer’s instructions. Briefly, an oligo-dT primer with an Illumina-compatible sequence was hybridized to RNA for reverse transcription. Second-strand synthesis was initiated using a random primer with an Illumina-compatible linker. The double-stranded library was purified and amplified via adapter addition, followed by purification. High-throughput single-end 75-bp sequencing was performed using NextSeq 550 (Illumina, San Diego, CA, USA). Then, Pearson’s correlation was used for gene co-expression analysis using the RNA expression data from Seoul National University Hospital (SNUH) PDAC samples. Correlation coefficient > 0.5 and *P* value < 0.05 indicated significant correlation. Gene Ontology (GO) and Kyoto Encyclopedia of Genes and Genomes pathway analyses were performed using the DAVID Bioinformatics Resources 6.8 database (https://david.ncifcrf.gov/). All analyses were performed using R version 3.5.1. (R Foundation for Statistical Computing, Vienna, Austria).

### siRNA transfection

siRNA targeting SLC5A3 (siSLC5A3) was purchased from Santa Cruz Biotechnology (Dallas, TX, USA). Cell transfection was performed using Lipofectamine RNAiMAX (Invitrogen), according to the manufacturer’s protocol. The cells were harvested 48–72 h post-transfection for subsequent analysis.

### Immunoblotting assay using antibodies

Protein lysates (30 µg) were prepared using the radioimmunoprecipitation assay lysis buffer (Rockland, Philadelphia, PA, USA) supplemented with Halt Protease and Phosphatase Inhibitor Cocktail (Thermo Fisher) and 0.5 M EDTA (Thermo Fisher). Protein concentrations were quantified using the Bio-Rad Protein Assay Kit (Bio-Rad, Hercules, CA, USA). Proteins were separated via sodium dodecyl sulfate-polyacrylamide gel electrophoresis (8–15%) and transferred onto polyvinylidene fluoride membranes (Merck Millipore). The membranes were blocked with 5% skim milk in Tris-buffered saline containing 0.5% Tween-20 for 1 h at approximately 22–25 °C, followed by overnight incubation with primary antibodies at 4 °C. The membranes were further incubated with horseradish peroxidase-conjugated secondary antibodies for 1 h at room temperature. Immunoblot signals were detected using the Clarity Western ECL Substrate (Bio-Rad) and visualized using ImageQuant LAS 4000 (GE Healthcare, Chicago, IL, USA).

The following antibodies were used in this study: Anti-cleaved/pro-caspase (CASP)-9 (sc-56076), anti-cleaved/pro-CASP3 (#9662/9661), anti-Bcl2 (#509), anti-CDK4 (#601), anti-CDK6 (sc-7961), anti-cyclin E (sc-481), anti-NRF2 (sc-365949), anti-GPX4 (sc-166570), anti-glyceraldehyde 3-phosphate dehydrogenase (sc-365062), anti-DRP1 (sc-101270), anti-OPA1 (sc-393296), and anti-VDAC1 (sc-390996) antibodies (all from Santa Cruz), anti-E-cadherin (610181) and anti-N-cadherin (610920) antibodies (all from BD Biosciences), anti-cyclin D1 (2922S), anti-Bax (2772T), anti-LC3B (2775S), anti-PUMA (4976S), and anti-parkin (4211S) antibodies (all from Cell Signaling Technology), anti-SLC5A3 (ab110368), anti-ribonucleotide reductase catalytic subunit M1 (RRM1; ab137114), anti-Ki67 (ab15580), and anti-BrdU (ab6326) antibodies (all from Abcam), horseradish peroxidase-conjugated goat anti-mouse (7076S) and goat anti-rabbit (7074S) secondary antibodies (all from Cell Signaling Technology), Alexa Fluor 488 goat anti-mouse IgG (H + L) (A-11001), Cy5 goat anti-rabbit IgG (H + L) (A-10523), and Alexa Fluor 488 goat anti-rat IgG (H + L) (A-11006) secondary antibodies (all from Invitrogen).

### Statistical analyses

Statistical analyses were conducted using one-way or two-way analysis of variance with GraphPad Prism version 10.3 (GraphPad Software). Data are represented as the mean ± standard deviation. Statistical significance was set at *P < 0.05, **P < 0.01, and ***P < 0.001.

## Results

### SLC5A3 levels are highly upregulated in patients with PDAC

**Fig. 1:**
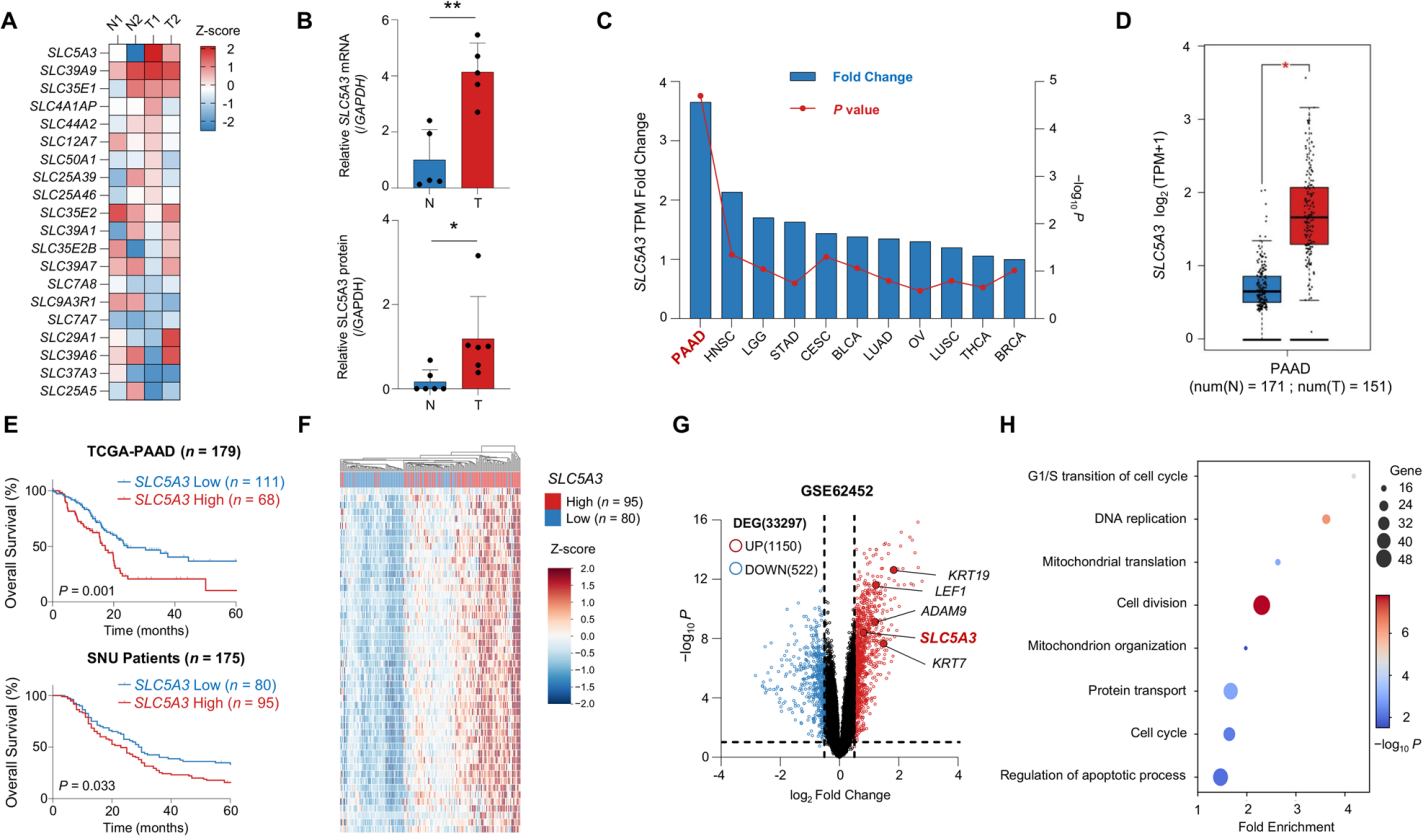
Solute carrier family 5 member 3 (SLC5A3) levels are highly upregulated and associated with poor prognosis in patients with pancreatic ductal adenocarcinoma (PDAC). **A** Heatmap showing the expression levels of SLC family genes in PDAC and adjacent normal pancreatic tissues determined from the RNA-sequencing (RNA-seq) data of patient tissues at the Gangnam Severance Hospital. **B** Bar graphs showing the SLC5A3 mRNA and protein levels in normal (N) (*n* = 5) and tumor (T) tissues (*n* = 5). **C** Comparative analysis of SLC5A3 expression levels in various cancer types. PAAD Pancreatic adenocarcinoma, HNSC Head and neck squamous cell carcinoma, LGG Brain lower grade glioma, STAD Stomach adenocarcinoma, CESC Cervical squamous cell carcinoma and endocervical adenocarcinoma, BLCA Bladder urothelial carcinoma, LUAD Lung adenocarcinoma, OV Ovarian serous cystadenocarcinoma, LUSC Lung squamous cell carcinoma, THCA Thyroid carcinoma, BRCA Breast invasive carcinoma. **D** Boxplot illustrating the SLC5A3 expression levels in normal (N) (*n* = 171) and tumor (T) tissues (*n* = 151) in The Cancer Genomic Atlas (TCGA) + Genotype-Tissue Expression (GTEx) dataset. **E** Kaplan–Meier overall survival curves for TCGA–pancreatic adenocarcinoma (PAAD) (*n* = 179) and Seoul National University (SNU) patient cohorts (*n* = 175). Cut-off determination was performed using X-tile software. **F** Clustered heatmap for SLC5A3 high (*n* = 95) and low (*n* = 80) expression in the SNU patient cohort (*n* = 175). The color scale represents the relative expression level of each gene. **G** Volcano plot showing the differentially expressed genes (DEGs) between normal (*n* = 61) and PDAC tissues (*n* = 69) in the GSE62452 dataset at |log2 FC| ≥ 0.5 and *P* < 0.05. Red dots indicate the upregulated genes, whereas blue dots indicate the downregulated genes. **H** Gene Ontology (GO) analysis of genes exhibiting strong correlations with SLC5A3 (*R* > 0.5; *P* < 0.05; false-discovery rate [FDR] < 0.05). Bubble plot shows the enrichment of biological processes associated with SLC5A3. *P*-values were calculated using the binomial test, and bubble size indicates the number of genes involved in each process. Statistical analyses were conducted using two-tailed unpaired Student’s *t* test in (**B** and **D**). (*P < 0.05; **P < 0.01).

We examined the expression levels of SLC family genes in PDAC using RNA-seq data from patient tissues and compared the gene expression levels between normal and tumor tissues (Fig. 1A). Among the analyzed SLC genes, levels of *SLC5A3* were the most significantly upregulated in PDAC tumor tissues compared to those in the adjacent normal tissues. To confirm this result, we performed additional analyses. SLC5A3 mRNA and protein levels were consistently elevated in tumor tissues, suggesting that SLC5A3 may play a role in PDAC tumorigenesis (Fig. 1B). Subsequently, we explored whether SLC5A3 overexpression is unique to pancreatic adenocarcinoma (PAAD) or observed in other cancer types. Data from the GEPIA2 and Human Protein Atlas datasets showed that SLC5A3 levels were elevated in multiple cancer types, with the most significant overexpression observed in PAAD (Fig. 1C) [27, 28]. This suggests that SLC5A3 contributes to the aggressive nature of pancreatic cancer. To validate these findings, we used larger datasets from The Cancer Genomic Atlas (TCGA) and Genotype-Tissue Expression (GTEx) databases, which confirmed the significant upregulation of SLC5A3 levels in PAAD tumor tissues compared to those in normal tissues (Fig. 1D) [29–31]. Next, we examined SLC5A3 levels across progressive stages. SLC5A3 levels are consistently upregulated in advanced stages, suggesting its role in disease progression (Fig. S1A). Survival analysis of multiple patient cohorts, including TCGA–PAAD and SNU cohorts, revealed that high SLC5A3 expression was associated with significantly poor overall survival, highlighting its potential as a prognostic marker for pancreatic cancer (Figs. 1E and S1B). To determine the functional implications of SLC5A3 overexpression, we performed gene expression profiling of the SNU patient cohort. Clustered Heatmap analysis revealed distinct differences in the gene expression patterns of patients with PDAC with high and low SLC5A3 levels, indicating that SLC5A3 influences the expression of specific genes involved in PDAC progression (Fig. 1F). Furthermore, SLC5A3 was among the most upregulated genes in PDAC, as shown in the volcano plot comparing normal and tumor tissues, further confirming its role in PDAC progression. (Fig. 1G). GO analysis of genes highly correlated with SLC5A3 expression using RNA-seq data from the SNU cohort indicated that SLC5A3 is associated with various critical biological processes, such as cell cycle regulation, mitochondrial function, and apoptosis (Fig. 1H) [32]. Collectively, these findings suggest that SLC5A3 plays multifaceted roles in PDAC progression by regulating the key processes essential for tumor growth and survival, underscoring the potential of targeting SLC5A3 as a novel therapeutic strategy.

### SLC5A3 promotes gemcitabine resistance and correlates with poor survival in patients with PDAC

**Fig. 2:**
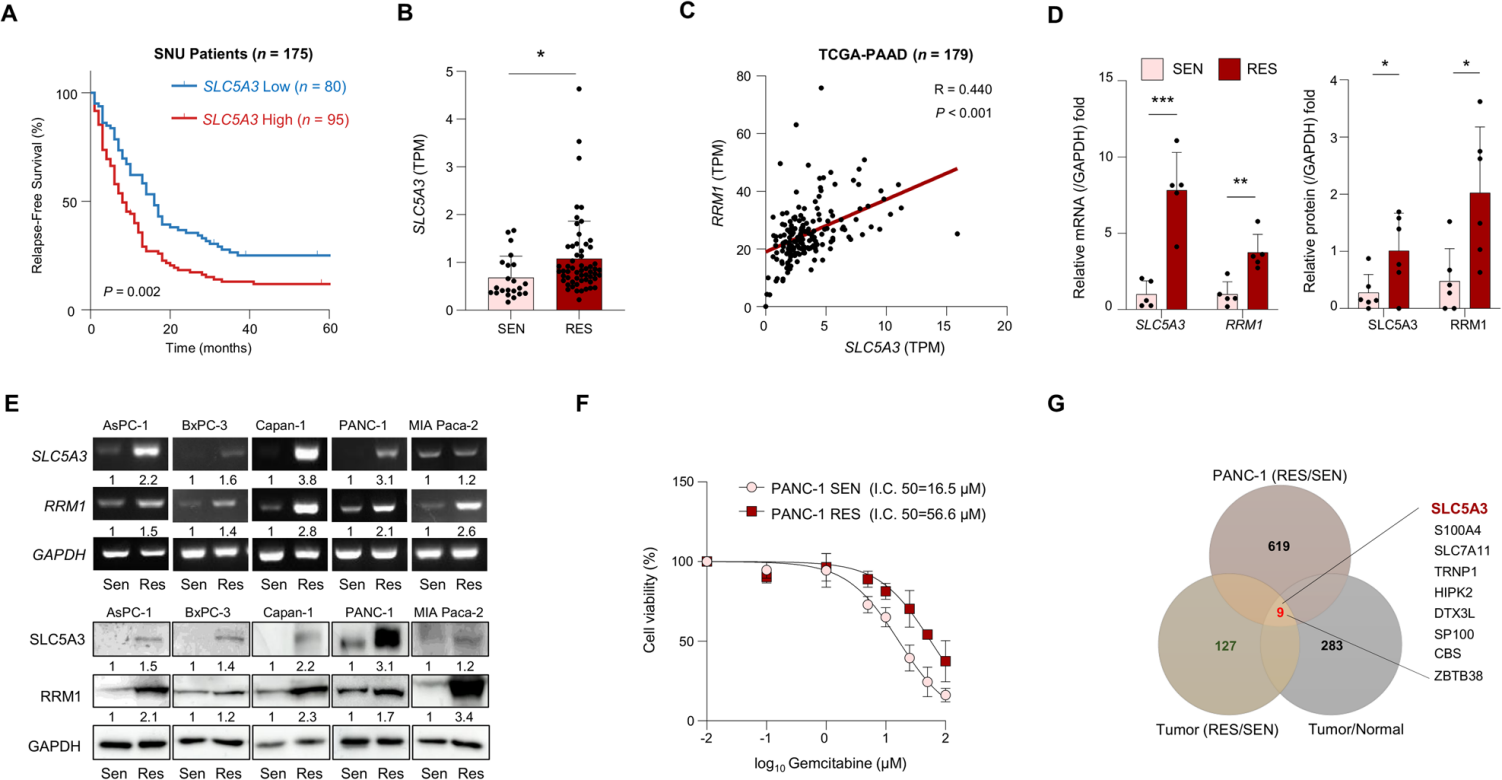
SLC5A3 promotes gemcitabine resistance and correlates with poor survival in patients with PDAC. **A** Kaplan–Meier survival curve showing the relapse-free survival (RFS) in the SNU patient cohort (*n* = 175). **B** Box plot showing the SLC5A3 expression levels in gemcitabine-sensitive (SEN) (*n* = 22) and -resistant (RES) PDAC tissues (*n* = 58). **C** Scatter plot showing the positive correlation between SLC5A3 and ribonucleotide reductase catalytic subunit M1 (RRM1) expression levels in TCGA–PAAD cohort (*n* = 179). **D** Bar graphs depicting the relative mRNA and protein levels of SLC5A3 and RRM1 in gemcitabine-sensitive (SEN) (*n* = 6) and -resistant (RES) PDAC tissues (*n* = 6). **E** Representative images of SLC5A3 and RRM1 expression levels in various PDAC cell lines (AsPC-1, BxPC-3, Capan-1, PANC-1, and MIA PaCa-2). **F** Cell viability assay showing the half-maximal inhibitory concentration (IC_50_) value of gemcitabine in PANC-1 cells. **G** Venn diagram illustrating the overlap of upregulated genes in three key categories: (1) Genes upregulated in PANC-1 RES cells, (2) genes upregulated in gemcitabine-resistant (GR) PDAC tumor tissues, and (3) genes upregulated in PDAC tumor tissues vs. normal tissues. Nine common genes, including *SLC5A3*, were upregulated in all three categories (fold-change > 2.0; *P* < 0.05). Statistical analyses were conducted using two-tailed unpaired Student’s *t* test in (**B** and **D**). (*P < 0.05; **P < 0.01; ***P < 0.001).

Next, we examined the survival outcomes in the SNU cohort to investigate the relationship between SLC5A3 expression and gemcitabine resistance. Patients with high SLC5A3 expression exhibited significantly short relapse-free survival, suggesting that elevated SLC5A3 expression is associated with gemcitabine resistance and tumor recurrence (Fig. 2A). To further analyze the relationship between SLC5A3 expression and gemcitabine resistance, we compared the SLC5A3 expression levels between gemcitabine-sensitive (SEN) and gemcitabine-resistant (RES) patients, which were categorized based on recurrence within six months post-treatment. SLC5A3 levels were significantly higher in the RES group than in the SEN group (Fig. 2B). This finding confirmed that elevated SLC5A3 expression is associated with increased gemcitabine resistance in PDAC. As RRM1 is a well-known marker of gemcitabine resistance, we further investigated the relationship between SLC5A3 and RRM1 expression levels (Fig. 2C) [33]. Among all tested resistance markers, RRM1 exhibited the strongest positive correlation with SLC5A3 in TCGA–PAAD cohort, making it the focus of subsequent tests. To further explore the role of SLC5A3 in gemcitabine resistance, we examined its mRNA and protein levels in SEN and RES PDAC tissues. SLC5A3 and RRM1 levels were higher in RES tissues than in SEN tissues (Fig. 2D). Next, we analyzed expression levels across several PDAC cell lines (AsPC-1, BxPC-3, Capan-1, PANC-1, and MIA PaCa-2) (Fig. S1C, D) and found that SLC5A3 and RRM1 were consistently overexpressed in RES cells compared to SEN cells (Fig. 2E). This consistent pattern across multiple cell lines indicated the broad conserved role of SLC5A3 in gemcitabine resistance. Cell viability assays were performed to quantify the extent of gemcitabine resistance. RES PANC-1 cells exhibited significantly higher IC_50_ values than the SEN PANC-1 cells, confirming enhanced resistance to gemcitabine in RES cells (Fig. 2F). To further understand the molecular mechanisms underlying gemcitabine resistance, we performed comparative gene expression analysis of PANC-1 RES cells, RES PDAC tissues, and general PDAC tissues. Venn diagram revealed nine common genes, including *SLC5A3*, upregulated in all three categories (Fig. 2G). These findings highlight the key roles of SLC5A3 in gemcitabine resistance and PDAC progression, reinforcing its potential as a therapeutic target for gemcitabine-resistant PDAC.

### SLC5A3 knockdown induces cell cycle arrest and inhibits proliferation of gemcitabine-resistant PDAC cells

**Fig. 3:**
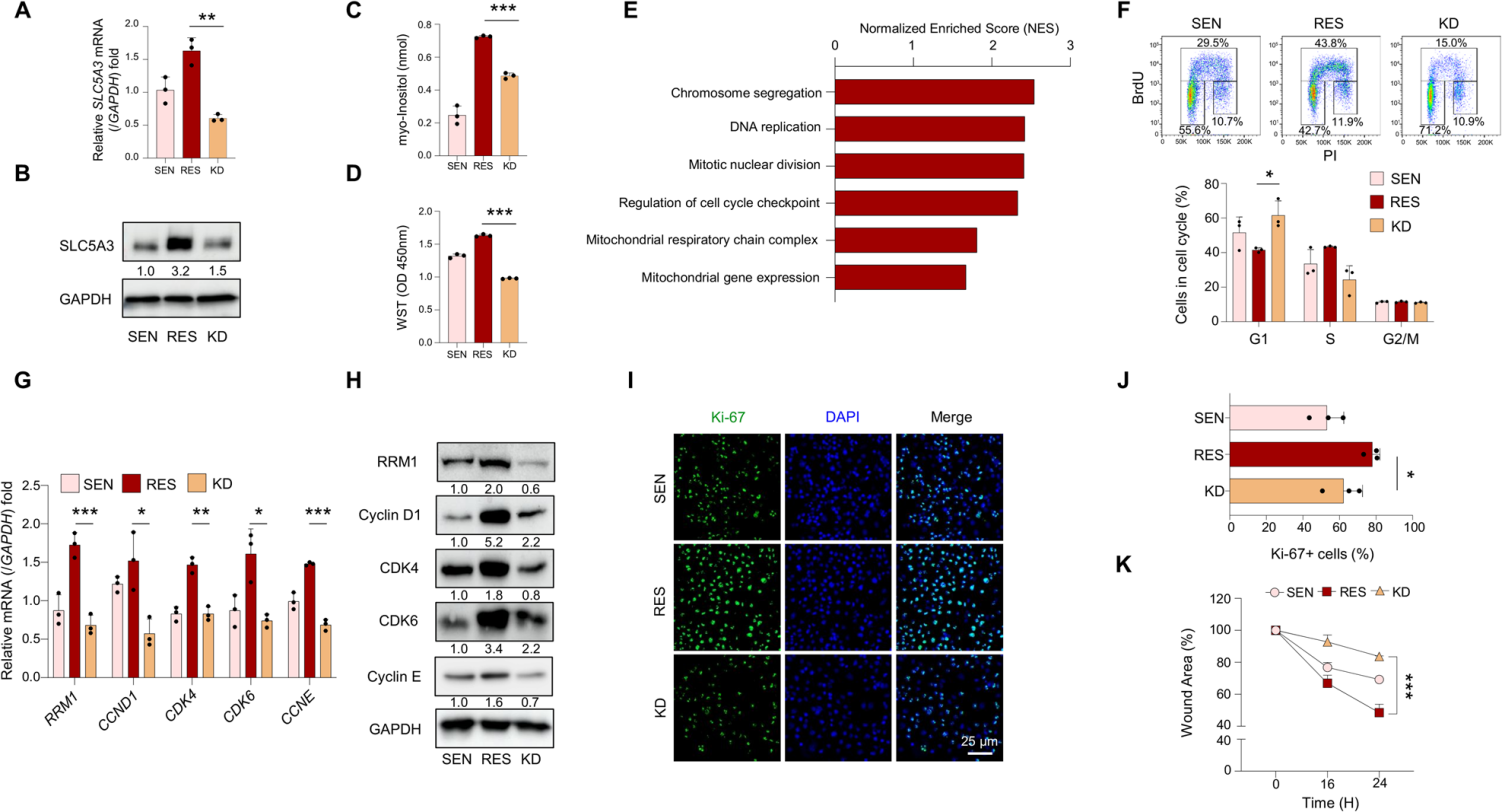
SLC5A3 knockdown (KD) induces cell cycle arrest and inhibits proliferation in gemcitabine-resistant PDAC cells. The KD cells were generated by transfecting RES cells with SLC5A3-specific siRNA, while RES cells transfected with non-targeting scrambled siRNA served as the control group. **A**, **B** mRNA (**A**) and protein (**B**) expression analyses showing the extent of SLC5A3 knockdown in RES cells. **C** Quantification of the intracellular myo-inositol levels in SEN, RES, and SLC5A3 KD cells. **D** Water-soluble tetrazolium salt (WST) assay showing the relative viability of SEN, RES, and SLC5A3 KD cells. **E** Gene set enrichment analysis (GSEA) showing the pathways enriched in RES cells compared to those in SLC5A3 KD cells. **F** BrdU incorporation analysis of cell cycle distribution in SEN, RES, and SLC5A3 KD cells. **G**, **H** mRNA (**G**) and protein (**H**) expression analyses of cell cycle regulatory genes in SEN, RES, and SLC5A3 KD cells. **I**, **J** Immunofluorescence staining for Ki-67 (**I**) in SEN, RES, and SLC5A3 KD cells (scale bar = 25 μm), and bar graph (**J**) showing the percentage of Ki-67-positive cells in each group. **K** Wound healing assay revealing the migration capacities of SEN, RES, and SLC5A3 KD cells. Data are represented as the mean standard deviation (S.D.) of three independent experiments (*n* = 3). Statistical analyses were conducted using ANOVA followed by Tukey’s multiple comparison test. (*P < 0.05; **P < 0.01; ***P < 0.001).

Considering the critical role of SLC5A3 in chemoresistance, we hypothesized that SLC5A3 knockdown (KD) significantly impairs cell survival and proliferation. We performed SLC5A3 knockdown in RES cells using siRNA specific to SLC5A3. The RES group, treated with non-targeting scrambled siRNA, served as the negative control to evaluate the specific effects of SLC5A3 knockdown. To validate the knockdown efficiency, we observed a marked reduction in SLC5A3 mRNA and protein levels in KD cells compared to the control group (Fig. 3A, B). Given that SLC5A3 functions as a myo-inositol transporter, we further measured intracellular myo-inositol levels [34, 35]. SLC5A3 KD significantly decreased the myo-inositol levels (Figs. 3C and S2A). Next, we assessed cell viability using the WST assay. Cell viability was significantly higher in RES cells than in SEN cells, but SLC5A3 KD significantly decreased the cell viability (Fig. 3D). As a myo-inositol transporter, SLC5A3 regulates osmotic stress and cellular metabolism, particularly in cancer cells [30]. Reduction in myo-inositol levels and downregulation of SLC5A3 and RRM1 mRNA levels in KD cells (Fig. S2B) suggest that the disruption of myo-inositol transport impairs the pathways essential for cell survival in RES cells. Next, gene set enrichment analysis (GSEA) was used to identify the biological pathways regulated by SLC5A3 [36]. GSEA revealed the significant enrichment of pathways involved in chromosome segregation, DNA replication, and cell cycle checkpoint regulation in RES cells compared to that in SLC5A3 KD cells (Fig. 3E). Therefore, SLC5A3 regulated critical processes, such as cell cycle progression and genomic stability, in RES cells. To assess the impact of SLC5A3 on cell cycle progression, we performed flow cytometry to analyze cell cycle distribution. SLC5A3 KD triggered G1 phase arrest, increased the proportion of cells in the G0/G1 phase, and reduced the S phase population (Figs. 3F and S2C). To investigate the molecular mechanisms underlying cell cycle arrest, we determined the expression levels of key cell cycle regulatory genes. Expression levels of *CCND1*, *CDK4*, *CDK6*, and *CCNE* were significantly higher in RES cells than in SEN cells; however, SLC5A3 KD significantly downregulated these expression levels (Fig. 3G, H). Therefore, SLC5A3 promoted cell cycle progression by modulating the key cell cycle regulators. Next, we examined the effect of SLC5A3 on cell proliferation via Ki-67 immunofluorescence staining. Percentage of Ki-67-positive cells was significantly higher in RES cells than in SEN cells, but SLC5A3 KD significantly decreased the number of proliferating cells (Fig. 3I, J). These findings further confirm the role of SLC5A3 in driving the proliferation of resistant cells. Finally, we investigated the role of SLC5A3 in cell migration using a wound healing assay. RES cells exhibited enhanced migration compared to SEN cells, but SLC5A3 KD significantly impaired wound closure (Figs. 3K and S2D, E). These results highlight the multifunctional roles of SLC5A3 in promoting cell survival, proliferation, and migration.

### SLC5A3 knockdown induces apoptosis and oxidative stress via mitochondrial dysfunction

**Fig. 4:**
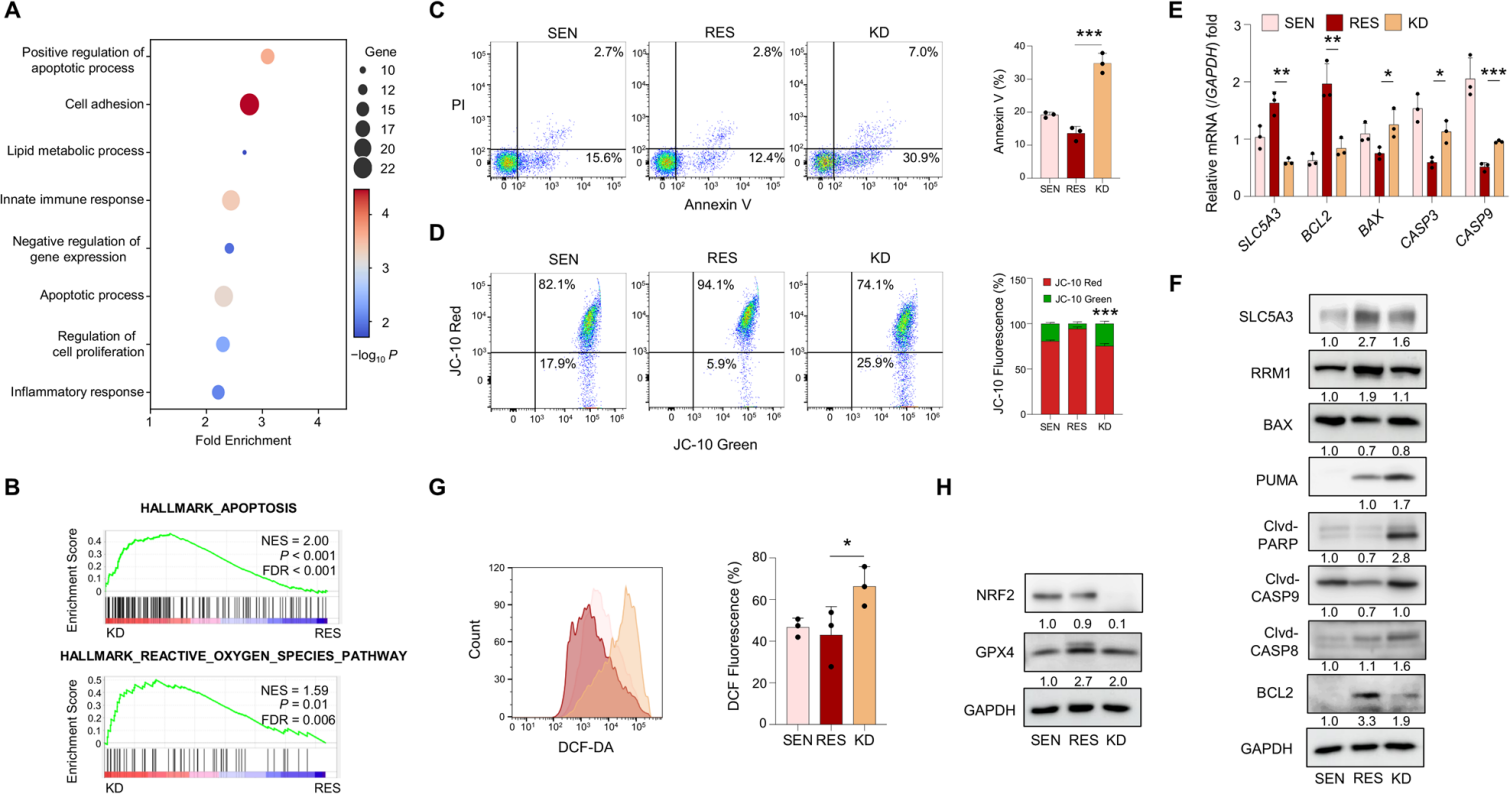
SLC5A3 KD induces apoptosis and oxidative stress via mitochondrial dysfunction. **A** Gene Ontology (GO) analysis of differentially expressed genes in SLC5A3 KD cells, with RES cells as the control group. **B** GSEA demonstrating the enrichment of apoptosis and reactive oxygen species (ROS) pathways in SLC5A3 KD cells compared to those in RES cells. **C** Flow cytometry analysis of apoptosis via Annexin V staining. **D** Assessment of mitochondrial membrane potential (MMP) via JC-10 staining. **E**, **F** mRNA (**E**) and protein (**F**) expression analyses of apoptotic markers in SEN, RES, and SLC5A3 KD cells. **G** Measurement of intracellular ROS levels via 2′,7′-dichlorofluorescein diacetate (DCF-DA) staining. **H** Protein expression analysis of antioxidant defense proteins. Data are represented as the mean S.D. of three independent experiments (*n* = 3). Statistical analyses were conducted using ANOVA followed by Tukey’s multiple comparison test. (*P < 0.05; **P < 0.01; ***P < 0.001).

We explored the biological effects of SLC5A3 KD on gemcitabine-resistant pancreatic cancer cells. RNA-seq data comparing SLC5A3 KD cells with RES cells were analyzed using GO analysis and GSEA to identify the affected pathways. GO analysis revealed significant upregulation of pathways related to apoptosis and oxidative stress regulation, suggesting that SLC5A3 plays key roles in modulating cell survival and stress responses (Fig. 4A). These findings were further supported by GSEA, which showed that both apoptotic and ROS pathways were upregulated in SLC5A3 KD cells (Fig. 4B). These results indicate that SLC5A3 deletion triggers apoptosis and oxidative stress via mitochondrial dysfunction and impaired antioxidant defense. To confirm apoptosis induction, we performed Annexin V staining to assess apoptotic cell death (Figs. 4C and S3A). Percentage of Annexin V-positive cells was significantly higher in SLC5A3 KD cells than in the SEN and RES cells, indicating robust induction of apoptosis following SLC5A3 depletion. Next, we examined the mitochondrial membrane potential via JC-10 staining. SLC5A3 KD cells showed significantly decreased mitochondrial membrane potential, indicating mitochondrial dysfunction, which is a hallmark of intrinsic apoptosis (Fig. 4D). At the molecular level, expression levels of key apoptotic markers were analyzed to verify these findings. SLC5A3 KD significantly decreased the levels of the anti-apoptotic gene, *BCL2*, and significantly increased those of pro-apoptotic genes, such as *BAX*, *CASP3*, and *CASP9* (Fig. 4E). Similar trends were observed at the protein level, with increased cleaved CASP9, CASP8, PARP, BAX and PUMA levels, further validating the activation of the intrinsic apoptotic pathway (Fig. 4F). GSEA revealed the activation of ROS-related pathways (Fig. 4B). Therefore, we measured the ROS levels in SLC5A3 KD cells. ROS production was significantly increased in KD cells compared to that in SEN and RES cells (Fig. 4G). This suggests that SLC5A3 depletion increases the oxidative stress, thereby inducing apoptosis, consistent with previous reports [37, 38]. To verify this, we examined the expression levels of key antioxidant defense regulators. NRF2 and GPX4, which are critical regulators of cellular antioxidant defense, were significantly downregulated in SLC5A3 KD cells (Fig. 4H). Reduction in antioxidant capacity further exacerbated oxidative stress and promoted cell death. Overall, SLC5A3 KD triggered apoptosis and oxidative stress by disrupting the mitochondrial function and downregulating the antioxidant pathways.

### Mitochondrial fragmentation and dysfunction induced by SLC5A3 depletion promote mitophagy and apoptosis

**Fig. 5:**
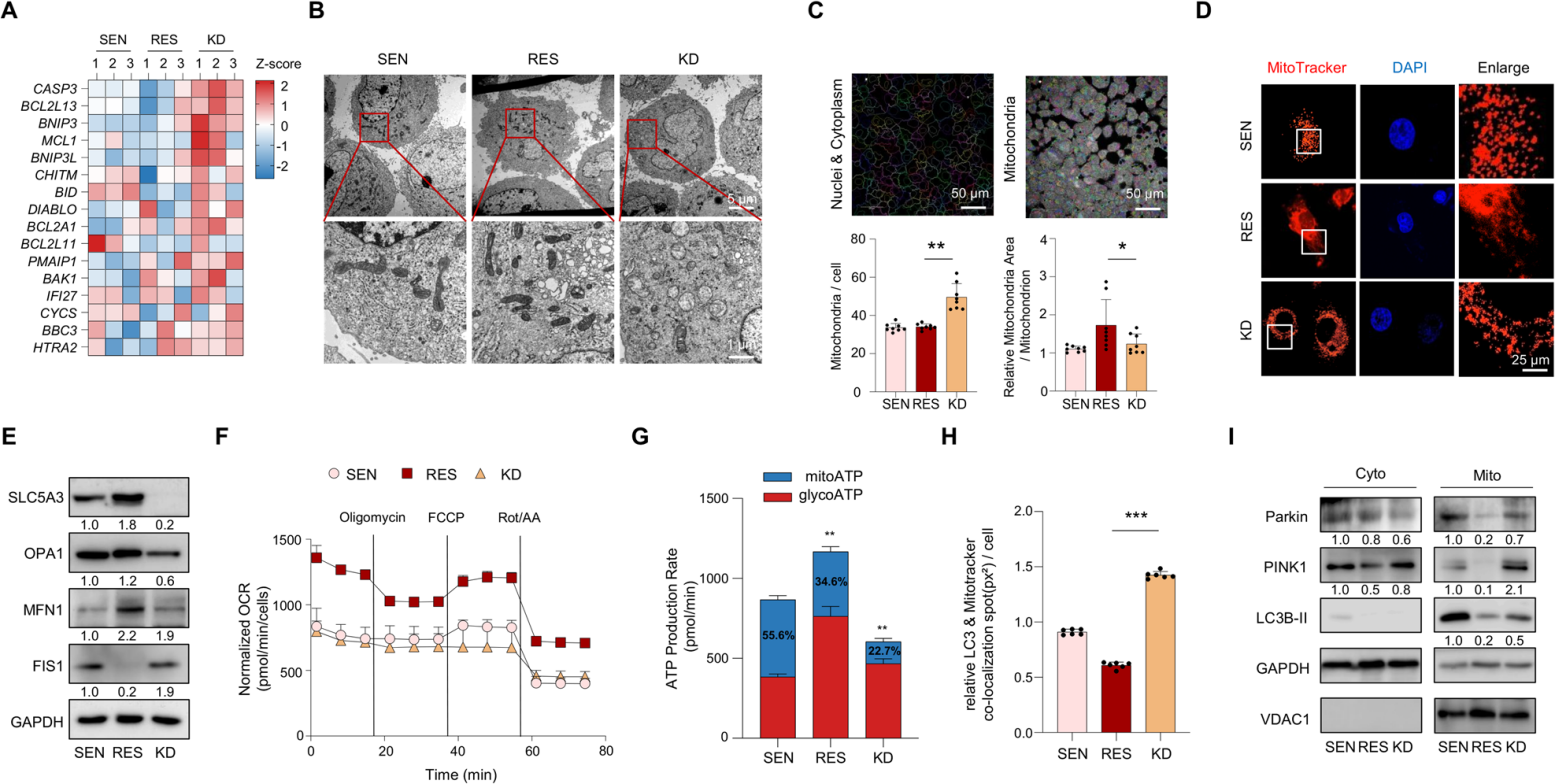
Mitochondrial fragmentation and dysfunction induced by SLC5A3 depletion promote mitophagy and apoptosis. **A** Heatmap showing the differential expression of apoptosis and mitochondrial regulation genes. **B** Transmission electron microscopy (TEM) images illustrating the increased mitochondrial fragmentation in KD cells. Scale bar = 5 μm (upper panels), 1 μm (lower panels). **C** High-content screening (HCS) analysis depicting the increased mitochondrial count and decreased mitochondrial area per cell in KD cells. Scale bar = 50 μm. **D** MitoTracker staining showing altered mitochondrial distribution in KD cells. Scale bar = 25 μm. **E** Protein expression analysis of mitochondrial dynamics-related proteins. **F** Oxygen consumption rate (OCR) analysis indicating reduced mitochondrial respiration in KD cells. **G** ATP production analysis showing decreased mitochondrial ATP production in KD cells. **H** Co-localization analysis of LC3 and MitoTracker revealed increased mitophagy in KD cells. **I** Protein expression analysis of the cytosolic (Cyto) and mitochondrial (Mito) fractions. Voltage-dependent anion channel 1 (VDAC1) and glyceraldehyde 3-phosphate dehydrogenase (GAPDH) were used as loading controls for the mitochondrial and cytosolic fractions, respectively. Data are represented as the mean S.D. of three independent experiments (*n* = 3). Statistical analyses were conducted using ANOVA followed by Tukey’s multiple comparison test. (*P < 0.05; **P < 0.01; ***P < 0.001).

Mitochondrial dynamics, including fission and fusion, are essential for cellular homeostasis, and any disruption of these processes can lead to mitochondrial dysfunction and apoptosis [39, 40]. SLC5A3 has been suggested to play critical roles in regulating these dynamics and maintaining the mitochondrial integrity [41, 42]. To verify this, we first examined the expression levels of key genes involved in apoptosis and mitochondrial regulation in SEN, RES, and KD cells. Heatmap revealed that the levels of pro-apoptotic genes, such as *CASP3*, *BAK1*, and *BNIP3*, were significantly upregulated, whereas those of anti-apoptotic genes, including *BCL2L13* and *MCL1*, were downregulated in SLC5A3-depleted cells (Fig. 5A). This finding indicates that SLC5A3 is crucial for maintaining mitochondrial structure and preventing apoptosis and that its depletion triggers apoptotic pathways. Next, we used TEM to visualize the structural changes in mitochondria caused by SLC5A3 depletion. TEM images showed that SLC5A3 depletion significantly increased mitochondrial fragmentation, leading to deformed mitochondrial morphologies such as rounded shape, disrupted cristae, and more vacuolization compared to RES group (Fig. 5B). The increase in fragmentation suggests that SLC5A3 depletion promotes mitochondrial fission, which is associated with cellular stress and mitochondrial dysfunction [43]. Furthermore, high-content screening revealed significantly increased number of mitochondria and decreased average mitochondrial area of KD cells (Fig. 5C) [44]. MitoTracker staining confirmed mitochondrial fission in SLC5A3-depleted cells, indicating the potential loss of mitochondrial function (Fig. 5D). Subsequently, we investigated the expression levels of key genes involved in mitochondrial dynamics at the molecular level. SLC5A3 depletion downregulated levels of the fusion-related proteins, OPA1 and MFN1, and upregulated the levels of the fission-related protein, FIS1 (Fig. 5E). These changes indicate that SLC5A3 maintains the balance between mitochondrial fusion and fission and that its depletion disrupts the balance. Consistent with these results, TCGA–PAAD cohort analysis revealed a positive correlation between SLC5A3 expression and mitochondrial fusion markers, *MFN1* and *OPA1* (Fig. S4A). To assess the functional consequences of these structural changes, we measured the OCR in SLC5A3-depleted cells. A significant decrease in OCR was observed, suggesting impaired mitochondrial respiration (Fig. 5F). This reduction in mitochondrial function was accompanied by markedly decreased mitochondrial ATP production and increased glycolytic ATP production in KD cells (Fig. 5G). This metabolic shift is common in cells undergoing mitochondrial dysfunction, as they shift from OXPHOS to glycolysis to meet their energy demands [45, 46]. Additionally, decrease in the extracellular acidification rate in SLC5A3 KD cells confirms that SLC5A3 is essential for maintaining mitochondrial bioenergetics (Fig. S4B). After observing mitochondrial dysfunction, we investigated whether mitophagy, the process by which cells remove damaged mitochondria, is activated in response to SLC5A3 depletion. Co-localization analysis of LC3B and MitoTracker showed significantly increased mitophagy in SLC5A3-depleted cells compared to that in the controls (Fig. 5H). Furthermore, expression levels of mitophagy markers, including PINK1, Parkin, and LC3B-II, were increased in the mitochondrial fractions of SLC5A3 KD cells (Fig. 5I). Although mitophagy typically removes the damaged mitochondria and maintains cellular homeostasis [11, 15], our data showed a significant reduction in the total mitochondrial mass via MitoTracker staining and electron microscopy analysis. This suggests that mitophagy not only targets the dysfunctional mitochondria but also depletes the healthy mitochondria. Furthermore, depletion of mitochondrial fusion markers combined with increased fission marker indicates the disruption of mitochondrial dynamics. Therefore, excessive activation of mitophagy in SLC5A3-depleted cells resulted in the loss of both healthy and damaged mitochondria, ultimately disrupting the cellular functions and promoting apoptosis.

### SLC5A3 depletion inhibits tumor growth and induces mitochondrial dysfunction in vivo

**Fig. 6:**
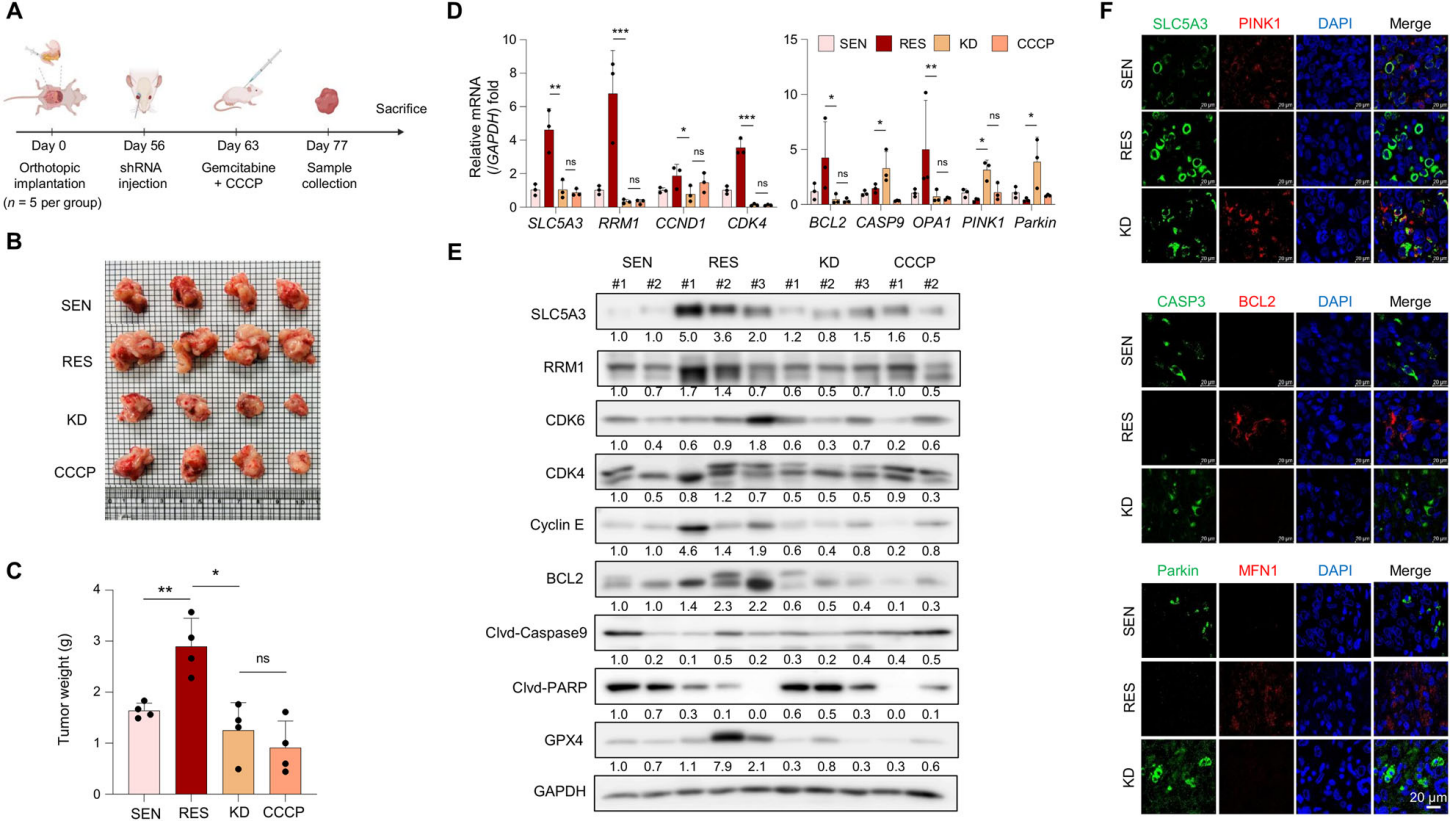
SLC5A3 depletion inhibits tumor growth and induces mitochondrial dysfunction in vivo. **A** Schematic representation of the orthotopic xenograft mouse model. PANC-1 cells were orthotopically implanted into the pancreas of BALB/c nude mice (*n* = 5 per group). SLC5A3 knockdown was performed by intravenously injecting shRNA lentiviral particles (shSLC5A3) or scramble shRNA particles eight weeks after tumor implantation. Gemcitabine and carbonyl cyanide 3-chlorophenylhydrazone (CCCP) were intraperitoneally injected twice per week. **B** Representative images of the harvested tumors from each group (SEN, RES, KD, and CCCP-treated). **C** Tumor weight measurements showing the significantly reduced tumor weights in KD and CCCP-treated groups compared to that in the RES group. **D** Quantitative analysis of the mRNA expression levels of key cell cycle regulators and mitochondrial/apoptotic genes in the tumors of each group. **E** Protein expression analysis of the cell cycle regulators, mitochondrial fusion proteins, and apoptotic markers in tumors. β-actin was used as a loading control. **F** Immunofluorescence staining of tumor sections for SLC5A3, PTEN-induced kinase 1 (PINK1), caspase 3 (CASP3), and BCL2. Scale bar = 20 μm. Data are represented as the mean S.D. of three independent experiments (*n* = 3). Statistical analyses were conducted using ANOVA followed by Tukey’s multiple comparison test. (*P < 0.05; **P < 0.01; ***P < 0.001; ns not significant).

To explore the effects of SLC5A3 depletion on tumor growth and mitochondrial function in gemcitabine-resistant pancreatic cancer, we used an orthotopic xenograft mouse model. BALB/c nude mice (five per group) were orthotopically implanted with PANC-1 SEN or RES cells (2.5 million cells per mouse). After eight weeks, SLC5A3 knockdown was performed by intravenously injecting shRNA lentiviral particles (shSLC5A3) while scramble shRNA particles were used as the control. Tumor growth was monitored weekly, followed by treatment with gemcitabine and CCCP, which is a mitochondrial uncoupler, to assess tumor growth and mitochondrial function (Fig. 6A). CCCP was used to investigate the broad effect of SLC5A3 on mitochondrial function, facilitating direct comparison with SLC5A3 depletion [47]. The harvested tumors and their corresponding weights showed that both SLC5A3 KD and CCCP treatment significantly reduced the tumor size and weight compared to those in the RES group (Fig. 6B, C). At both the protein and mRNA levels, SLC5A3 KD downregulated key cell cycle regulators, CDK4 and CCND1, but upregulated pro-apoptotic markers, such as BAX and CASP3, and mitochondrial regulators in the KD and CCCP-treated groups (Fig. 6D, E). The changes in gene expressions confirmed the crucial role of SLC5A3 in maintaining the mitochondrial integrity and supporting cell survival in gemcitabine-resistant tumors. Immunofluorescence staining also confirmed these observations, showing reduced PINK1, PARKIN, and BCL2 levels and significantly increased CASP3 levels in the KD and CCCP-treated groups (Fig. 6F). These results indicate that SLC5A3 depletion induces mitochondrial dysfunction and apoptosis. The similarities between the effects of SLC5A3 depletion and CCCP treatment emphasize the critical roles of mitochondrial dynamics in gemcitabine-resistant pancreatic cancer. In conclusion, our results suggest that SLC5A3 KD inhibits tumor growth by disrupting the mitochondrial function, inducing apoptosis, and arresting cell cycle progression. Therefore, strategies targeting SLC5A3 can be used as novel therapeutic approaches for gemcitabine-resistant pancreatic cancer by enhancing tumor suppression and alleviating mitochondrial dysfunction.

## Discussion

Various studies have demonstrated the mechanisms of gemcitabine resistance, including altered drug metabolism and activation of pro-survival pathways [48–50]. However, the complex nature of chemoresistance remains unclear, emphasizing the need to explore other cellular processes involved in chemoresistance. In this study, we identified SLC5A3 as a key factor promoting gemcitabine resistance in PDAC cells (Figs. 1 and 2). We found that SLC5A3 levels were significantly upregulated in gemcitabine-resistant PDAC cells compared to those in gemcitabine-sensitive cells. Moreover, SLC5A3 inhibition impaired the mitochondrial function, increased ROS production, and enhanced apoptosis. These findings suggest that SLC5A3 is critical for cancer cell survival under chemotherapeutic stress.

SLC5A3 inhibition not only triggered mitochondrial fission but also depleted the healthy mitochondria, reducing the overall mitochondrial function essential for energy production (Fig. 5). This finding was unexpected as SLC5A3 was previously shown to play key roles in sodium and inositol transport [21]. Mitochondrial fission often leads to mitophagy via the PINK1/Parkin pathway [51–53]. However, our findings indicate that SLC5A3 inhibition accelerates the removal of both damaged and healthy mitochondria, contributing to cellular energy imbalance and increased susceptibility to apoptosis (Fig. 4). Interestingly, mitophagy and similar disruptions in mitochondrial homeostasis have been associated with chemoresistance in other cancers, including ovarian and breast cancers [54, 55]. Our data highlight the potential of targeting mitophagy in the treatment of pancreatic cancer.

This study revealed a clear association between SLC5A3 expression and mitochondrial OXPHOS (Fig. 5F). In chemo resistant cancer cells, OXPHOS is often upregulated to meet the increased energy demand [56, 57]. Here, SLC5A3 inhibition disrupted OXPHOS, significantly decreasing the total ATP levels and disrupting energy homeostasis (Fig. 5G). ATP depletion not only reduces the cell capacity to perform essential functions but also increases cell vulnerability to apoptosis [58]. Disruption of ATP production exacerbates oxidative stress in cells as the lack of energy interferes with the ability of the cell to repair oxidative damage and maintain mitochondrial function [59]. Upon energy depletion, cells cannot sustain essential metabolic functions, leading to impaired mitochondrial repair and overall cell stability [60]. In this study, apoptosis was increased in SLC5A3-inhibited cells, supporting the critical role of SLC5A3 in the maintenance of mitochondrial function and cell survival. In addition to affecting the mitochondrial dynamics and OXPHOS, SLC5A3 inhibition affected cell cycle progression (Fig. 3). SLC5A3 inhibition partially arrested the cell cycle in gemcitabine-resistant PDAC cells, consistent with the decreased levels of cyclin D1 and CDK4/6 in previous reports [61, 62].

Mitochondrial homeostasis is maintained by the balance among mitochondrial biogenesis, fusion, fission, and mitophagy [63]. Accumulation of damaged mitochondria and the loss of functional mitochondria compromise cell survival [64, 65]. SLC5A3 plays a key role in maintaining mitochondrial homeostasis, particularly in chemoresistant cancer cells, by preventing excessive mitophagy and preserving the mitochondrial integrity. Further investigation into the mechanisms by which SLC5A3 interacts with key mitochondrial regulatory proteins, such as DRP1, OPA1, and MFN2, will provide deeper insights into its roles in modulating cellular dynamics and energy homeostasis in PDAC.

In conclusion, this study demonstrated that SLC5A3 is a critical regulator of mitochondrial dynamics, cell cycle progression, and chemoresistance in PDAC. By promoting mitochondrial stability, regulating cell cycle checkpoints, and inhibiting ROS-induced apoptosis, SLC5A3 promote gemcitabine resistance in PDAC cells. However, SLC5A3 inhibition impeded these effects, increasing apoptosis and sensitivity to chemotherapy in PDAC cells. Our findings suggest SLC5A3 as a promising therapeutic target in the treatment of gemcitabine-resistant PDAC.

## Supplementary information

Supplementary files

Supplementary Table

Original western blots

## Data Availability

Correspondence and requests for materials should be addressed to Joon Seong Park and Hyo Jung Kim.
