# Supplementary material for: SLC5A3 depletion promotes apoptosis by inducing mitochondrial dysfunction and mitophagy in gemcitabine-resistant pancreatic cancer cells
Source: Cell Death Dis. 2025 Mar 7;16(1):161. doi: 10.1038/s41419-025-07476-5 (PMC11889219; doi:10.1038/s41419-025-07476-5)
Supplement: Supplementary file 1 — Supplementary files [file 41419_2025_7476_MOESM1_ESM.docx]

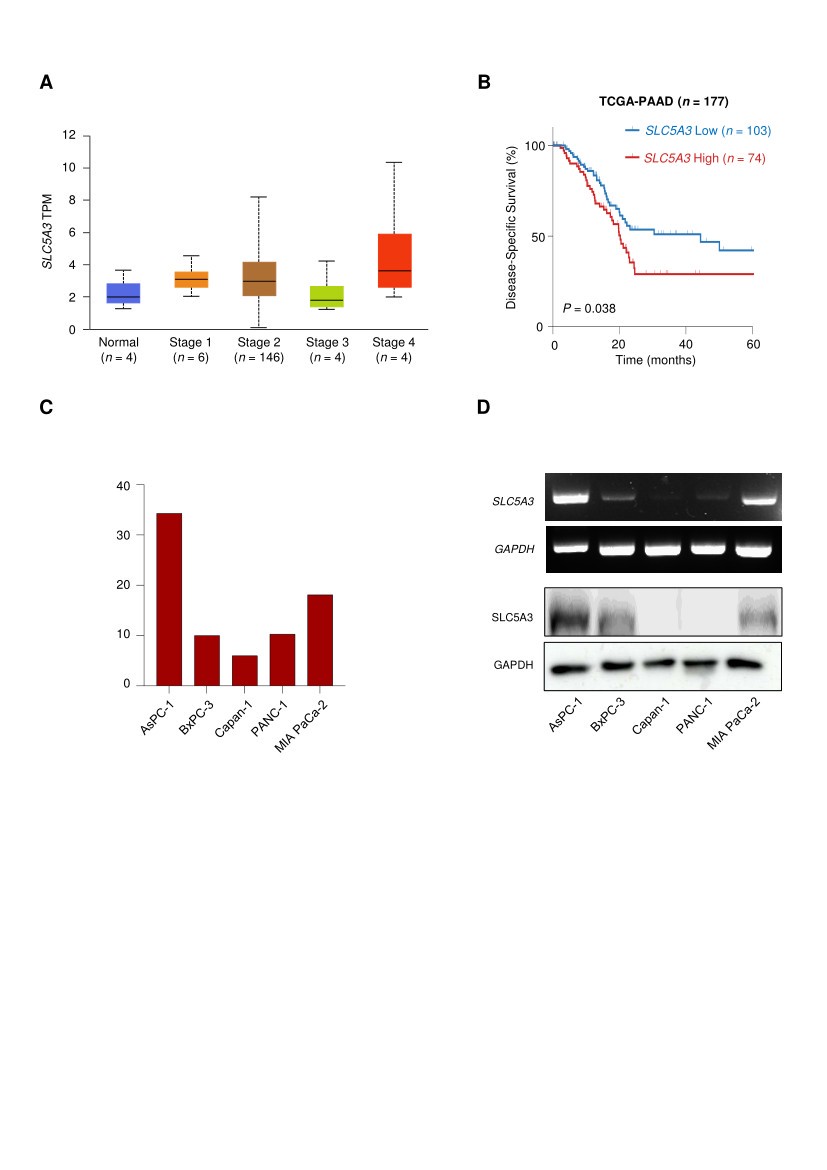


**Supplementary Fig. 1 *SLC5A3* expression levels in pancreatic ductal adenocarcinoma (PDAC). A** *SLC5A3* expression across different PDAC stages. **B** Kaplan–Meier survival curve shows the disease-specific survival in pancreatic adenocarcinoma (PAAD) patients separated by *SLC5A3* high (*n* = 74) and low (*n* = 103) groups. The dataset of PAAD patients is from The Cancer Genome Atlas (TCGA). **C** Bar graph represents SLC5A3 transcript per million (TPM) level in PDAC cell lines using The Human Protein Atlas data. **D** PCR and Western blot analysis of SLC5A3 expression in human PDAC cell lines, including AsPC-1, BxPC-3, Capan-1, PANC-1, and MIA PaCa-2 with GAPDH as a loading control. (Full and uncropped western blots can be found in original data files).


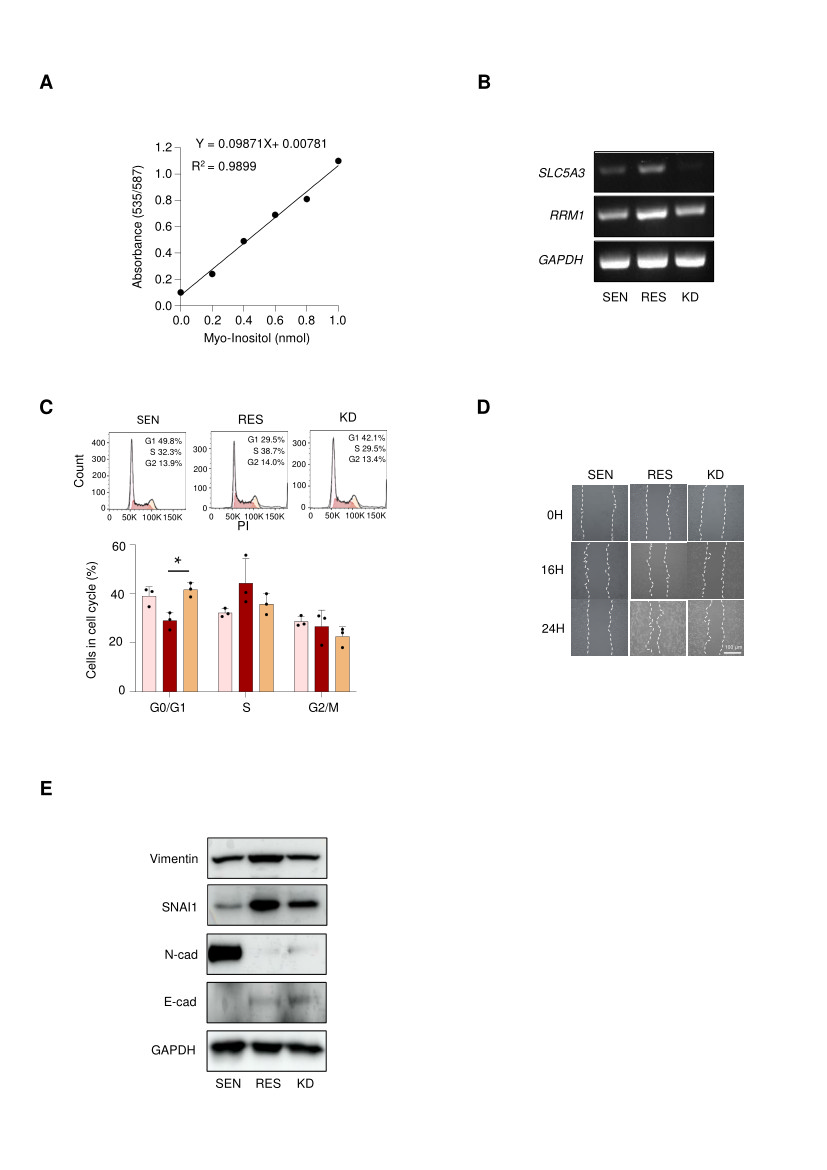


**Supplementary Fig. 2 Impact of SLC5A3 knockdown on mesenchymal markers and cell cycle. A** Myo-inositol absorbance levels (535/587 nm) measured in SEN, RES, and KD groups. **B** PCR of *SLC5A3* and *RRM1* expression in PANC-1 cells. **C** PI-based cell cycle analysis using flow cytometry to assess DNA content and cell cycle distribution. **D** Representative microscopy images displaying migration capacity in SEN, RES, and KD cells over 16 to 24 hours. **E.** Western blot analysis showing the expression of Vimentin, SNAI1, N-cadherin, and E-cadherin in PANC-1 cells with GAPDH as a loading control. Data are represented as the mean S.D. of three independent experiments (n = 3). Statistical analyses were conducted using ANOVA followed by Tukey’s multiple comparison test. (*P < 0.05).

**
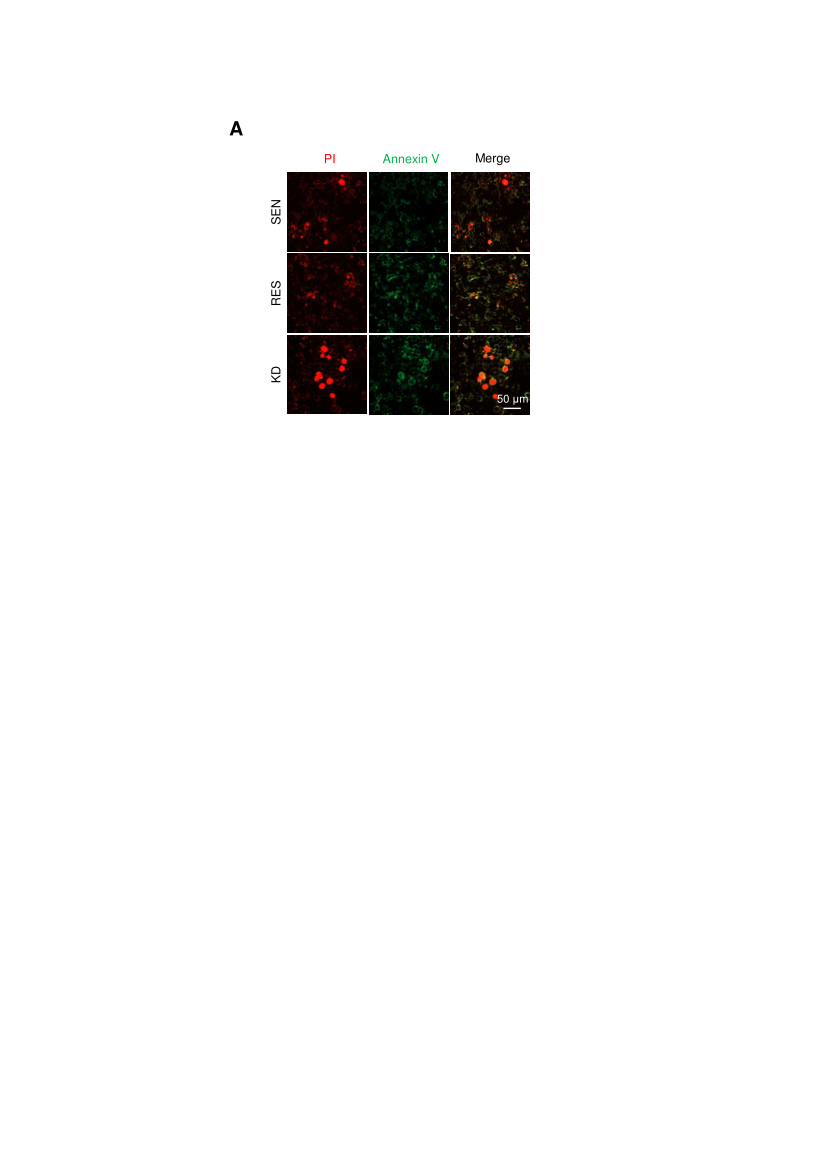
**

**Supplementary Fig. 3 Confocal image of Annexin V/PI staining in SEN, RES, and KD PDAC cells, indicating apoptosis rates across groups (scale bar = 50 μm).**


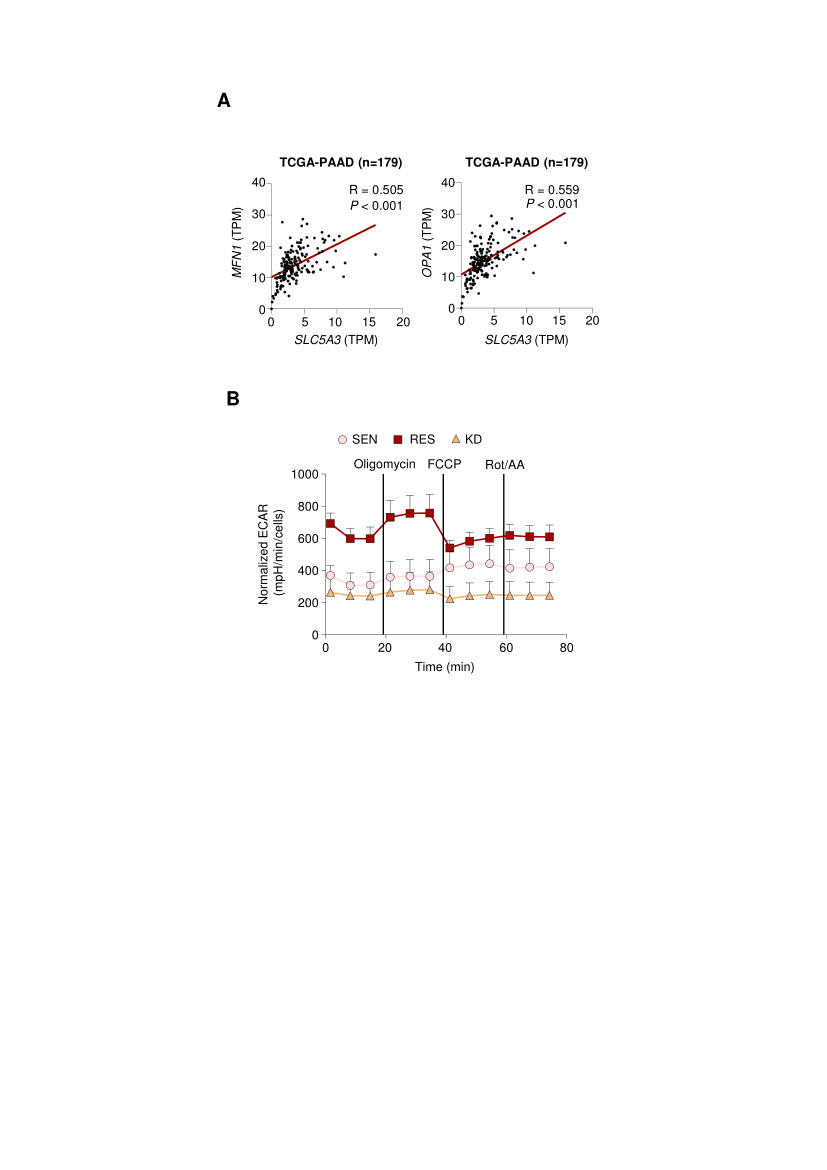


**Supplementary Fig. 4 Correlation of SLC5A3 with mitochondrial dynamics and metabolic profiles. A** Correlation of SLC5A3 expression with MFN1 and OPA1 levels in TCGA-PAAD (*n* = 179), showing significant correlations (R = 0.505 and 0.559, respectively; *P* < 0.001 for both). **B** Extracellular acidification rate (ECAR) over time in SEN, RES, and KD groups, showing changes following oligomycin, FCCP, and Rot/AA injections.
