# Supplementary material for: SLC5A3 depletion promotes apoptosis by inducing mitochondrial dysfunction and mitophagy in gemcitabine-resistant pancreatic cancer cells
Source: Cell Death Dis. 2025 Mar 7;16(1):161. doi: 10.1038/s41419-025-07476-5 (PMC11889219; doi:10.1038/s41419-025-07476-5)
Supplement: Supplementary file 2 — Supplementary Table [file 41419_2025_7476_MOESM2_ESM.docx]

**Supplementary Table. 1 Sequences of primers used in real-time RT-PCR.**

| Gene | Direction (5’ to 3’) | Sequences |
| --- | --- | --- |
| *SLC5A3* | F | GAG ATT GGC GGG TTT GAG GA |
|  | R | ATC TGT TGG ATT CCG CAG CA |
| *RRM1* | F | TCT CAG ACG GAA ACA GGC AC |
|  | R | GCA CAG GTT GCT GCA TTT GA |
| *GAPDH* | F | GTC TCC TCT GAC TTC AAC AGC G |
|  | R | ACC ACC CTG TTG CTG TAG CCA A |
| *CDK4* | F | GTG GCC CTC AAG AGT GTG AG |
|  | R | ATG TGG CAC AGA CGT CCA TC |
| *CDK6* | F | TCA CAC CGA GTA GTG CAT CG |
|  | R | ACT ATA GAT GCG GGC AAG GC |
| *CCND1* | F | GAC CCC GCA CGA TTT CAT TG |
|  | R | CAC AGA GGG CAA CGA AGG T |
| *CCNE1* | F | AGG GAG CGG GAT GCG A |
|  | R | TAT TGT CCC AAG GCT GGC TC |
| *CDH2* | F | CCT CCA GAG TTT ACT GCC ATG AC |
|  | R | GTA GGA TCT CCG CCA CTG ATT C |
| *VIM* | F | AGG CAA AGC AGG AGT CCA CTG A |
|  | R | ATC TGG CGT TCC AGG GAC TCA T |
| *BAX* | F | TCA GGA TGC GTC CAC CAA GAA G |
|  | R | TGT GTC CAC GGC GGC AAT CAT C |
| *BCL2* | F | ATG TGT GTG GAG AGC GTC AA |
|  | R | ACA GTT CCA CAA AGG CAT CC |
| *CASP3* | F | GGA AGC GAA TCA ATG GAC TCT GG |
|  | R | GCA TCG ACA TCT GTA CCA GAC C |
| *CASP9* | F | GTT TGA GGA CCT TCG ACC AGC T |
|  | R | CAA CGT ACC AGG AGC CAC TCT T |
| *FIS1* | F | GTA AAG GCA TCG TGC TGC TC |
|  | R | CTC GTA TTC CTT GAG CCG GT |
| *MFN1* | F | AAT GCT CAA AGG GTG CTC CT |
|  | R | GCA TTA TCT GGC GTT GCT GG |
| *MFN2* | F | GTC TGA CCT GGA CCA CCA AG |
|  | R | TGC AGT TGG AGC CAG TGT AG |
| *OPA1* | F | TGG AAT GAC TTT GCG GAG GA |
|  | R | GCT GCA TCC CAT TGC TGT TT |
| *DNM1L* | F | TCA CCC GGA GAC CTC TCA TT |
|  | R | TCT GCT TCC ACC CCA TTT TCT |
| (Mouse) *SLC5A3* | F | TCC ATC CTA AGG CGA ATG CC |
|  | R | ACC AGT ACC ATA CCG AGG CT |
| (Mouse) *GAPDH* | F | GGA GAG TGT TTC CTC GTC CC |
|  | R | ATG AAG GGG TCG TTG ATG GC |
| (Mouse) *CDK4* | F | TAC ATA CGC AAC ACC CGT GG |
|  | R | CAA CTG GTC GGC TTC AGA GT |
| (Mouse) *CCND1* | F | CCA ACA ACT TCC TCT CCT GCT |
|  | R | AGG GGG TCC TTG TTT AGC CA |
| (Mouse) *CASP3* | F | ATG GGA GCA AGT CAG TGG AC |
|  | R | GTC CAC ATC CGT ACC AGA GC |
| (Mouse) *PINK1* | F | GTG GGA CTC AGA TGG CTG TC |
|  | R | CGC TCT ACA CTG GAG CTG TT |
| (Mouse) *PRKN* | F | AAA TGC ATC TGG AGG GGA CG |
|  | R | TGG CTG CTT CTG AAT CCC TC |
