# Supplementary material for: SLC5A3 depletion promotes apoptosis by inducing mitochondrial dysfunction and mitophagy in gemcitabine-resistant pancreatic cancer cells
Source: Cell Death Dis. 2025 Mar 7;16(1):161. doi: 10.1038/s41419-025-07476-5 (PMC11889219; doi:10.1038/s41419-025-07476-5)

**Full uncropped original western blots for Figure 1**

Fig.1B-SLC5A3

**
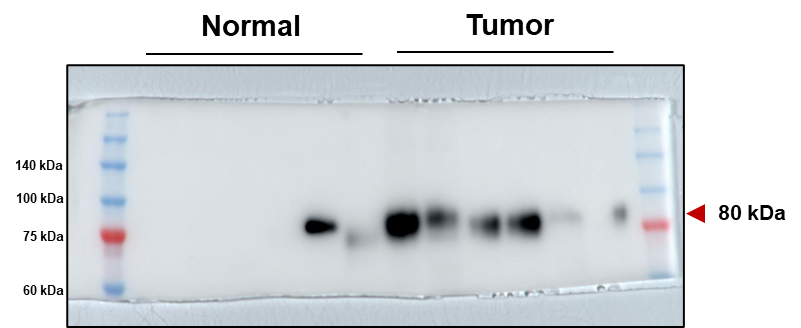
**

Fig.1B-GAPDH


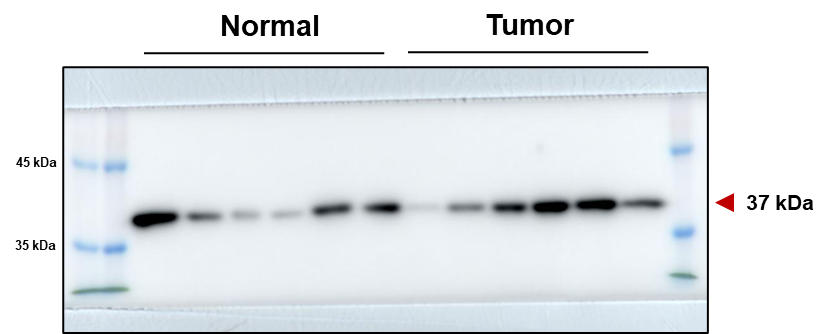


**Full uncropped original western blots for Figure 2**

Fig.2D-SLC5A3

**
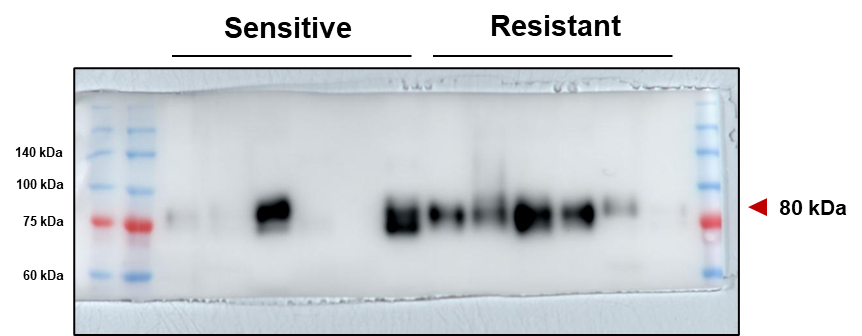
**

Fig.2D-RRM1


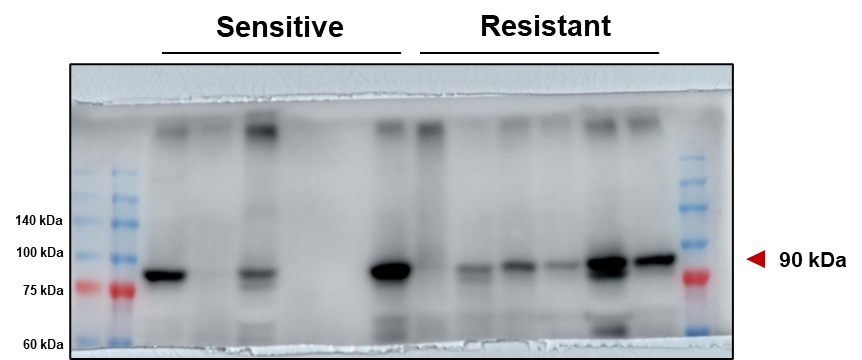


Fig.2D-GAPDH


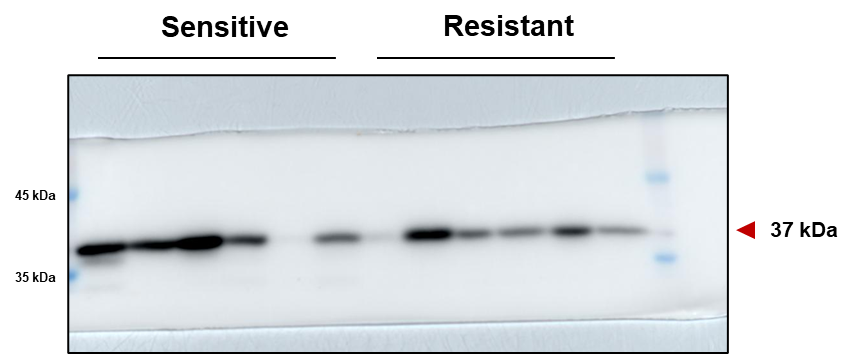


Fig.2E-SLC5A3


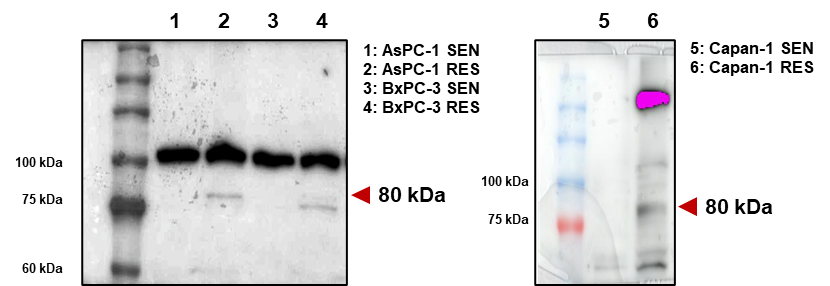


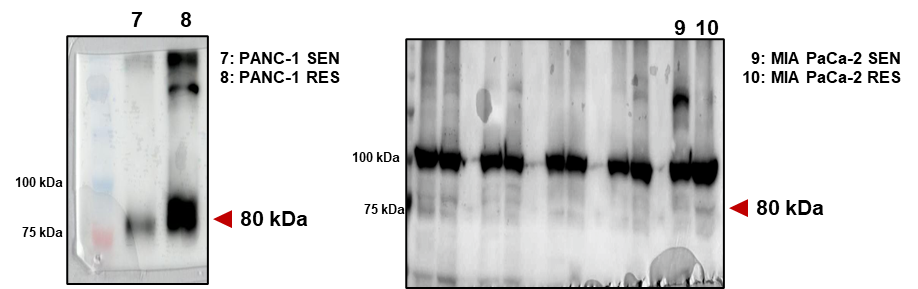


Fig.2E-RRM1


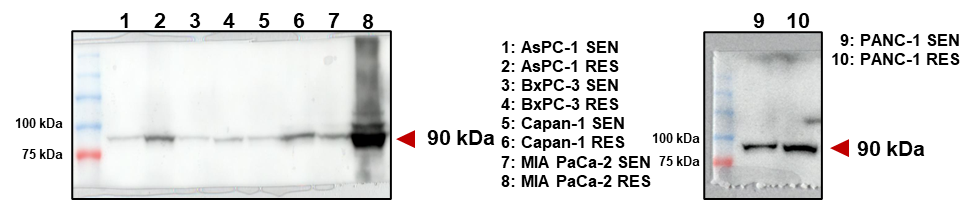


Fig.2E-GAPDH


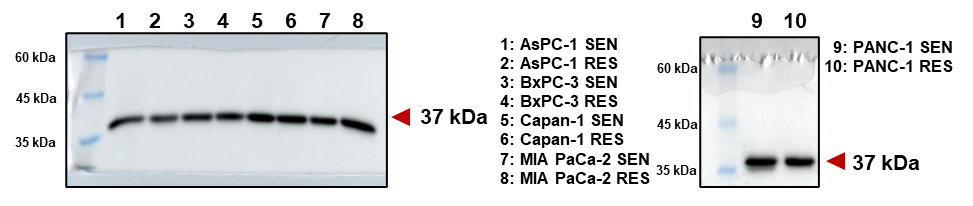


**Full uncropped original western blots for Figure 3**

Fig.3B-SLC5A3


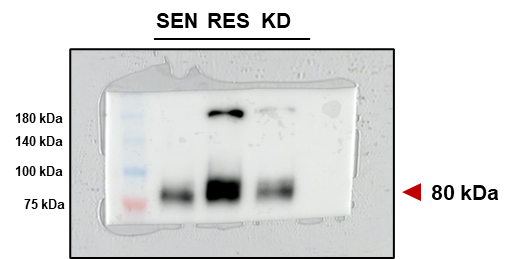


Fig.3B-GAPDH


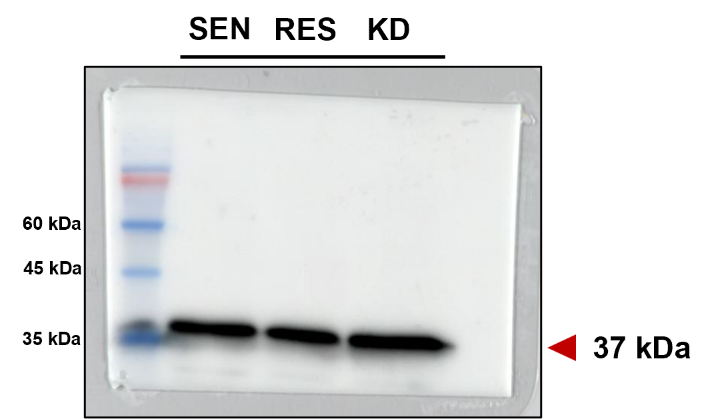


Fig.3F-RRM1


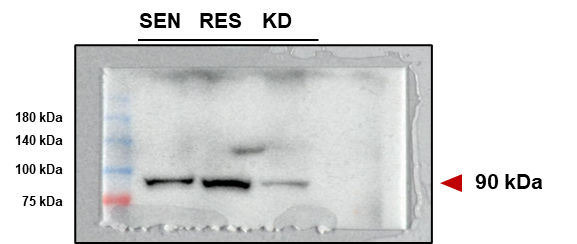


Fig.3F-Cyclin D1


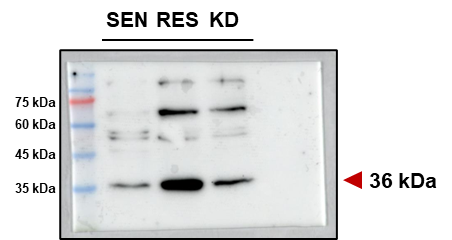


Fig.3F-CDK4


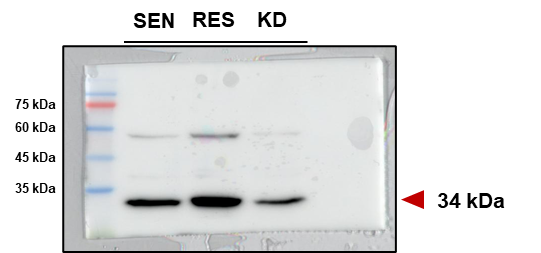


Fig.3F-CDK6


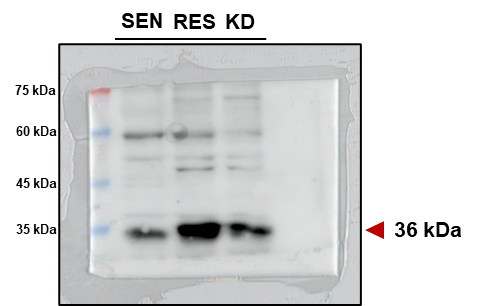


Fig.3F-Cyclin E1


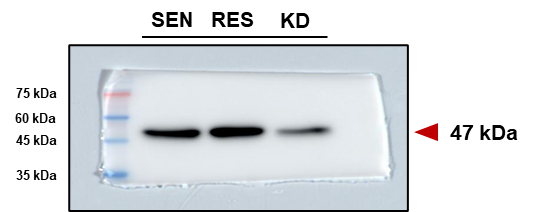


Fig.3F-GAPDH


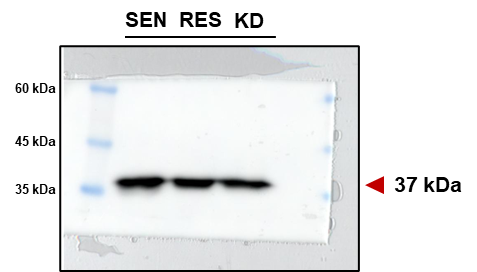


**Full uncropped original western blots for Figure 4**

Fig.4F-SLC5A3


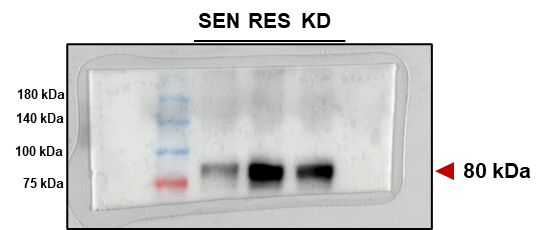


Fig.4F-RRM1


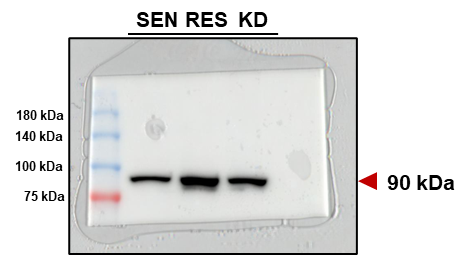


Fig.4F-BAX


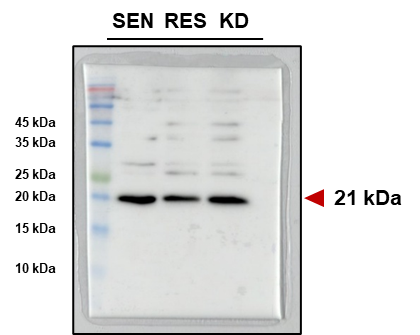


Fig.4F-PUMA


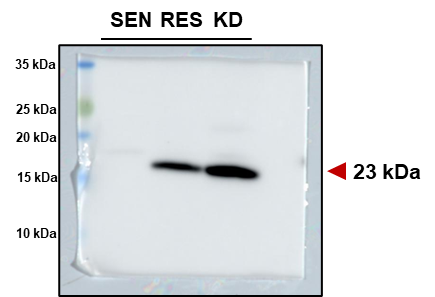


Fig.4F-Clvd-PARP


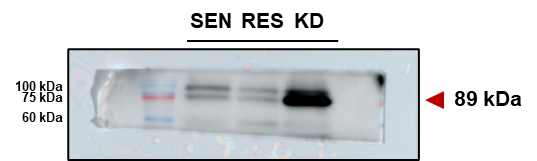


Fig.4F-Clvd-CASP9


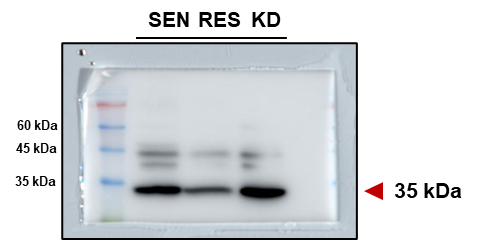


Fig.4F-Clvd-CASP8


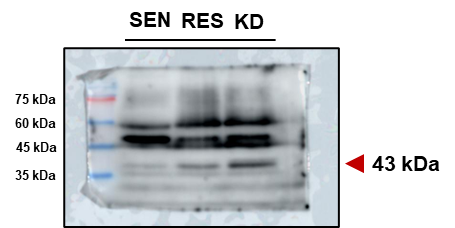


Fig.4F-BCL2


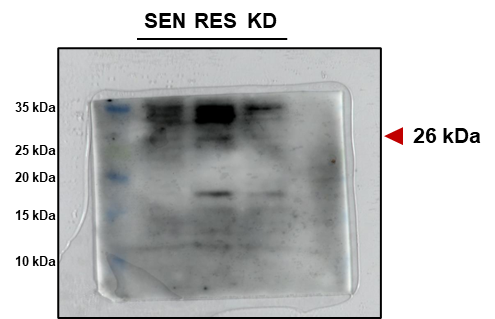


Fig.4F-GAPDH


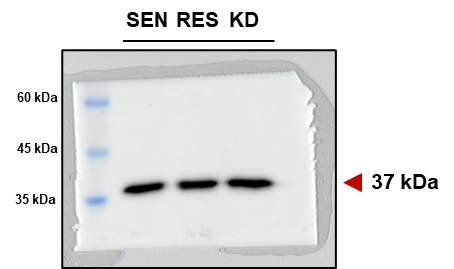


Fig.4H-NRF2


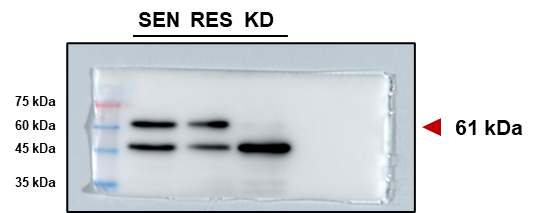


Fig.4H-GPX4


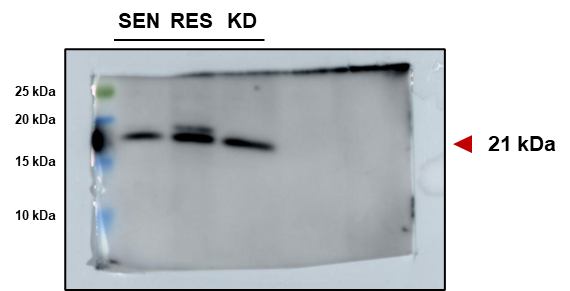


Fig.4H-GAPDH


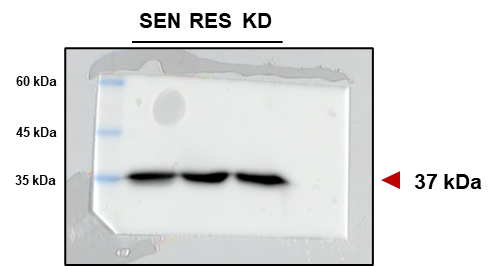


**Full uncropped original western blots for Figure 5**

Fig.5E-SLC5A3


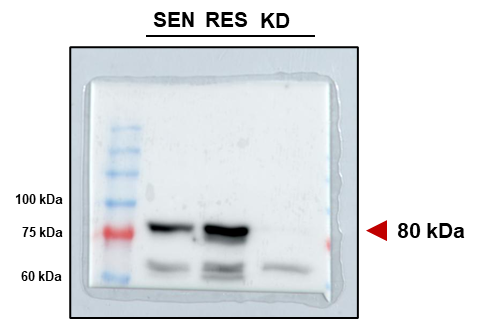


Fig.5E-OPA1


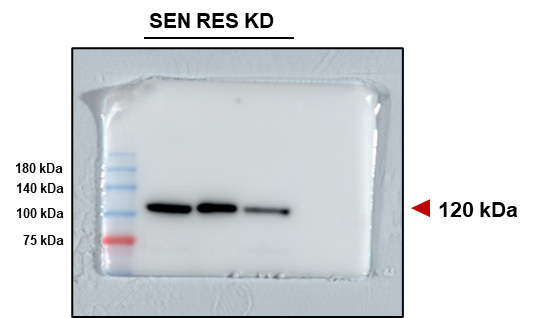


Fig.5E-MFN1


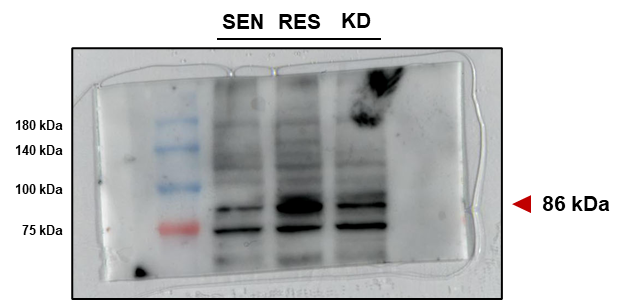


Fig.5E-FIS1


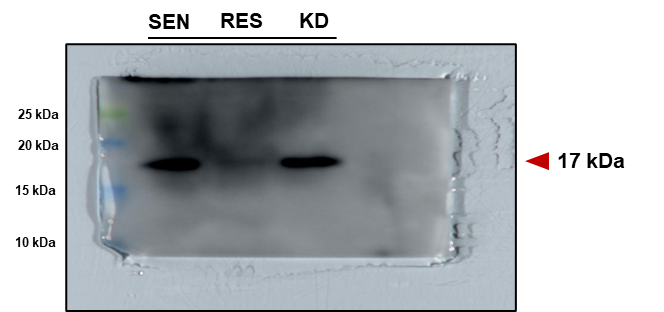


Fig.5E-GAPDH


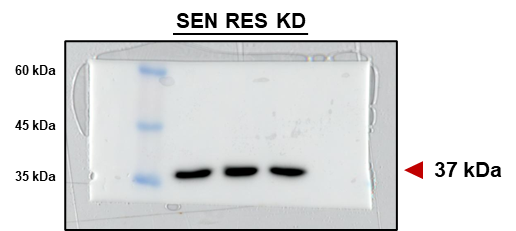


Fig.5I-Parkin


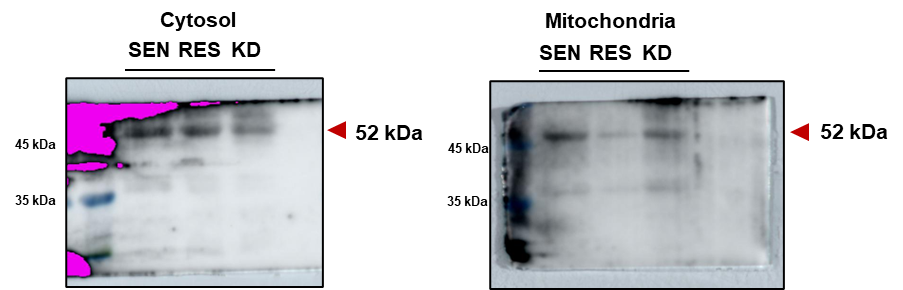


Fig.5I-PINK1


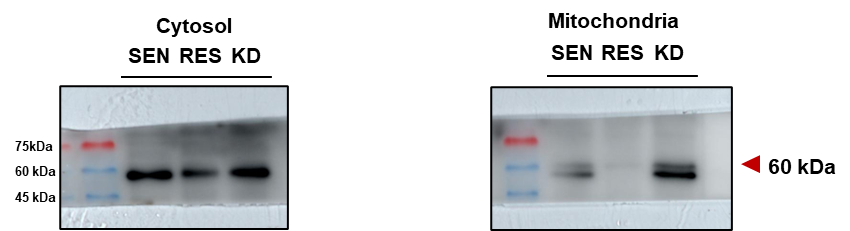


Fig.5I-LC3B-II


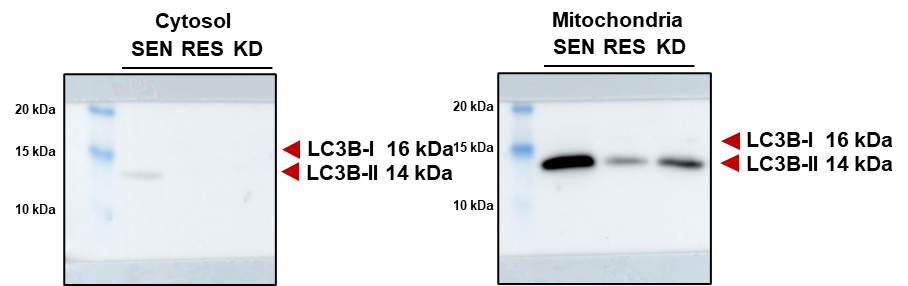


Fig.5I-GAPDH


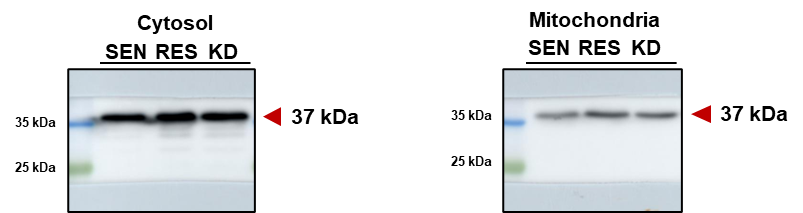


Fig.5I-VDAC1


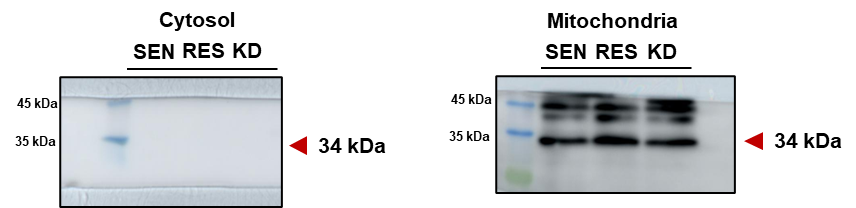


**Full uncropped original western blots for Figure 6**

Fig.6E-SLC5A3

**
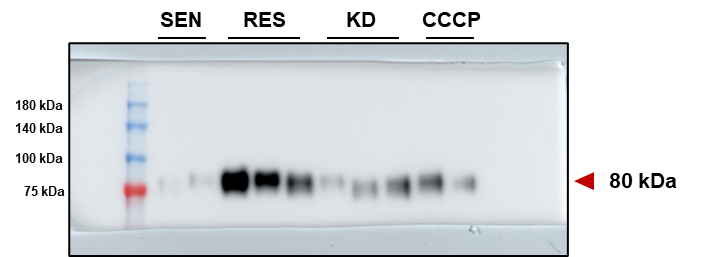
**

Fig.6E-RRM1


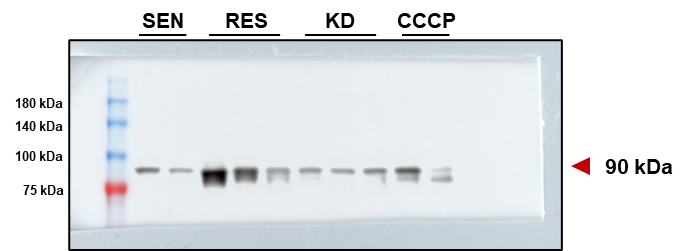


Fig.6E-CDK6


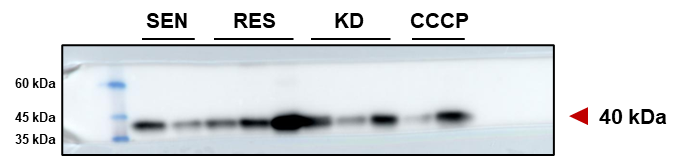


Fig.6E-CDK4


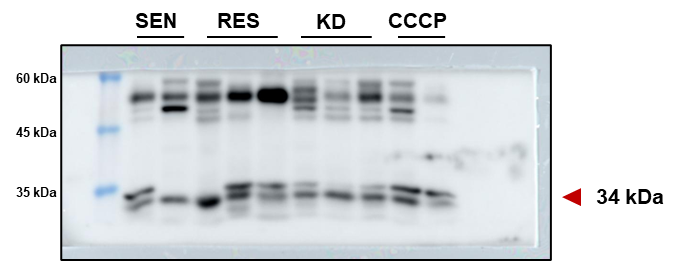


Fig.6E-Cyclin E


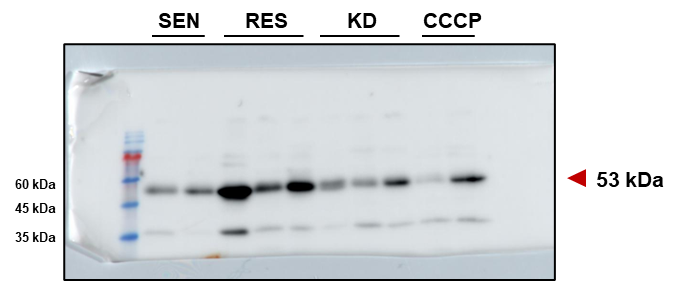


Fig.6E-BCL2


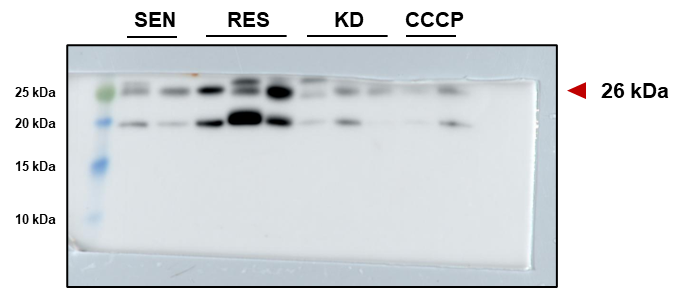


Fig.6E-Clvd-Caspase9


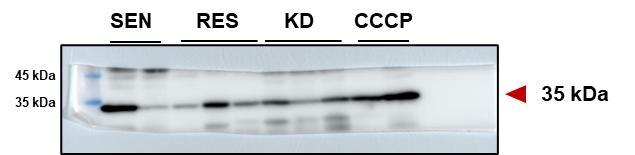


Fig.6E-Clvd-PARP


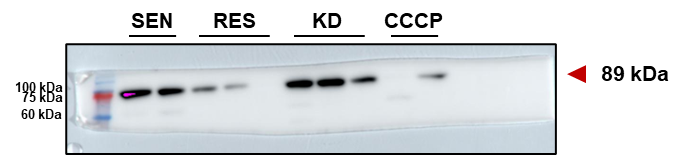


Fig.6E-GPX4


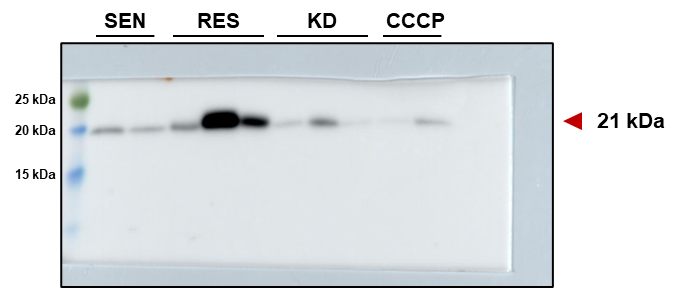


Fig.6E-GAPDH


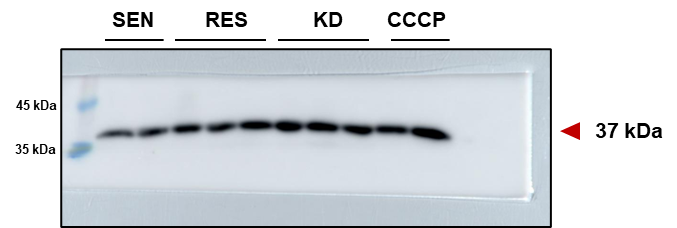


**Full uncropped original western blots for Supplementary Figure 1**

Fig.S1D-SLC5A3

**
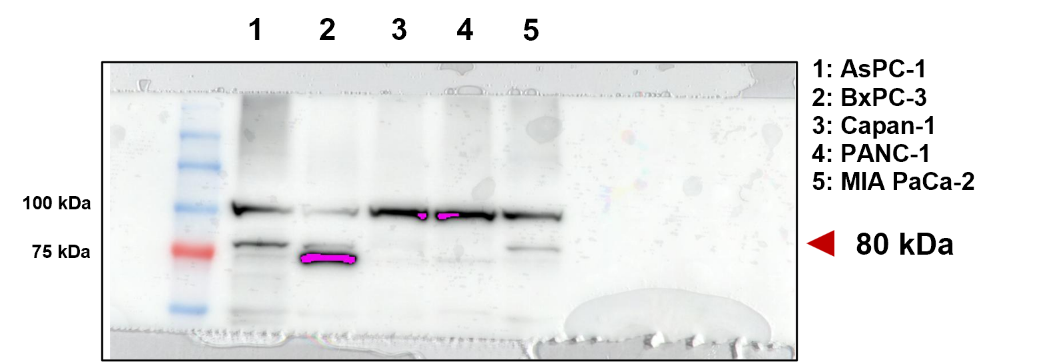
**

Fig.S1D-GAPDH

**
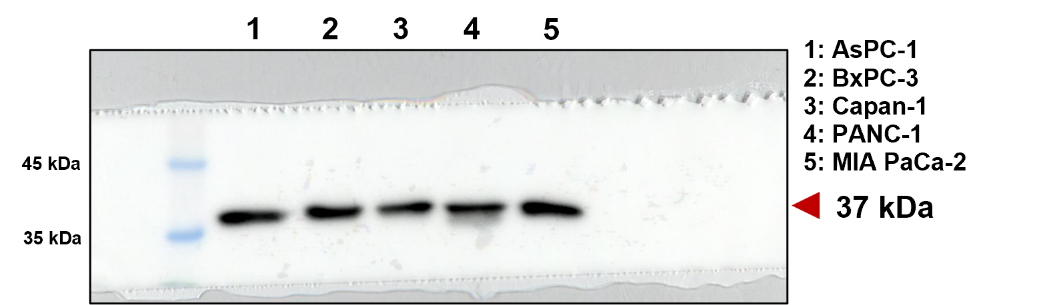
**

**Full uncropped original western blots for Supplementary Figure 2**

Fig.S2E-Vimentin


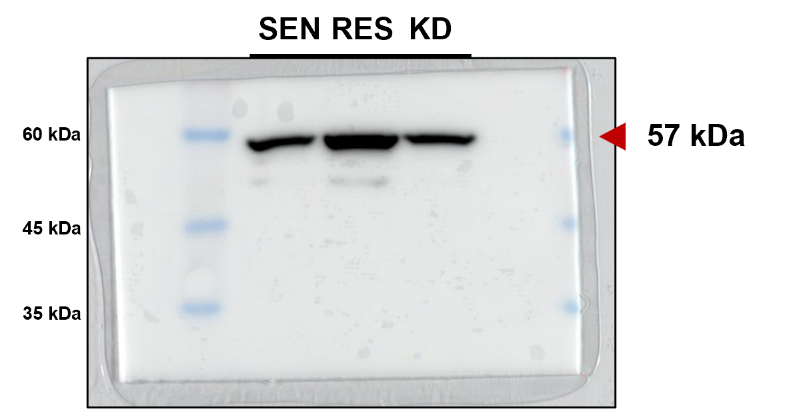


Fig.S2E-SNAI1


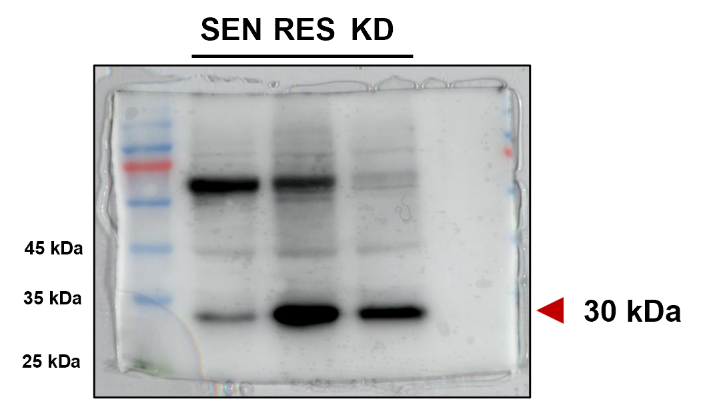


Fig.S2E-N-cadherin


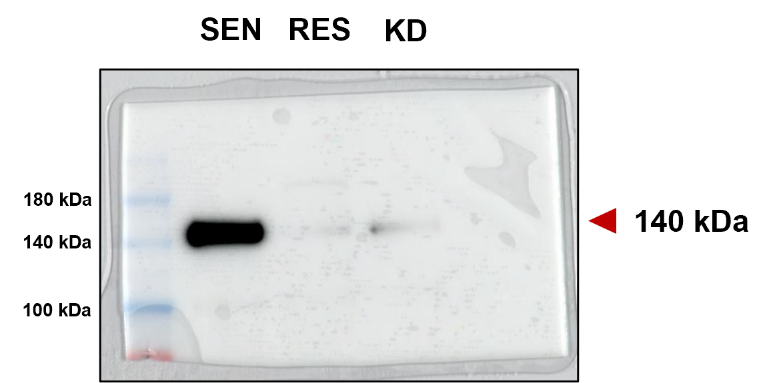


Fig.S2E-E-cadherin


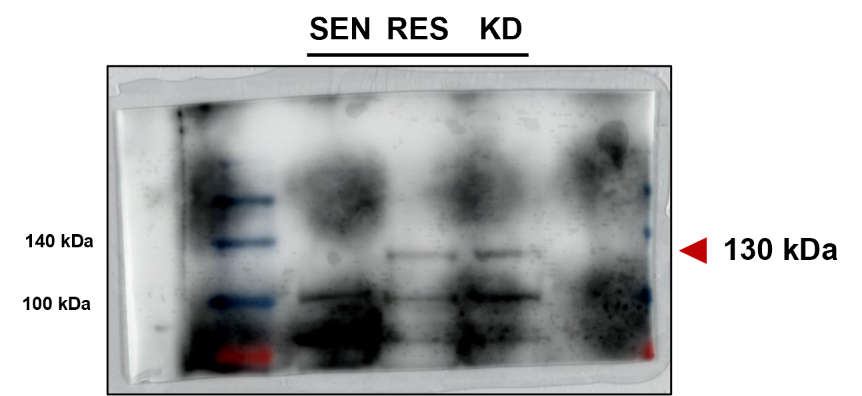


Fig.S2E-GAPDH


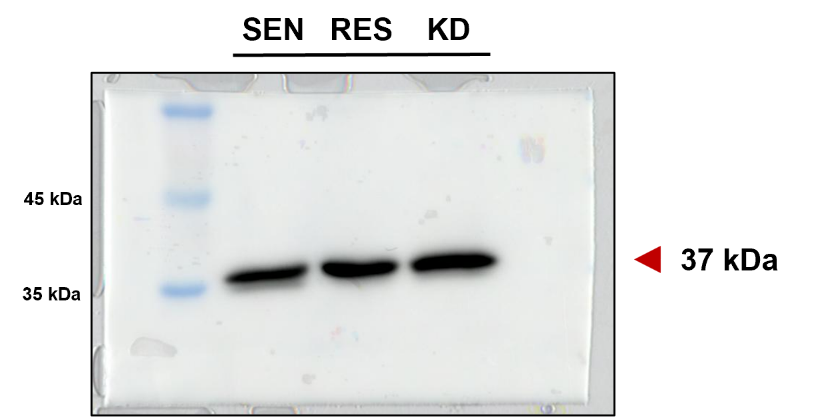

Supplement: Supplementary file 3 — Original western blots [file 41419_2025_7476_MOESM3_ESM.docx]
